# Three new species of *Creptotrema* (Trematoda, Allocreadiidae) with an amended diagnosis of the genus and reassignment of *Auriculostoma* (Allocreadiidae), based on morphological and molecular evidence

**DOI:** 10.1051/parasite/2021065

**Published:** 2021-10-13

**Authors:** Lidiane Franceschini, Aline Aguiar, Aline Cristina Zago, Priscilla de Oliveira Fadel Yamada, Mariana Bertholdi Ebert, Reinaldo José da Silva

**Affiliations:** 1 São Paulo State University (Unesp), Institute of Biosciences, Section of Parasitology Rua Professor Doutor Antônio Celso Wagner Zanin, n° 250 Botucatu São Paulo CEP 18618-689 Brazil; 2 São Paulo State University (Unesp), Institute of Biosciences, Department of Biodiversity Avenida 24A, 1515, Bela Vista Rio Claro São Paulo CEP 13506-900 Brazil

**Keywords:** Digeneans, Teleosts, Phylogeny, Host-parasite relationships, Neotropical region

## Abstract

Diversity of *Creptotrema* was investigated using morphological and molecular tools, including data for *Creptotrema creptotrema* (type-species). Three new species, parasites of Brazilian fishes, are described: *Creptotrema conconae* n. sp. (type-host, *Imparfinis mirini* Haseman), *Creptotrema schubarti* n. sp. (type-host, *Characidium schubarti* Travassos) and *Creptotrema megacetabularis* n. sp. (type-host, *Auchenipterus osteomystax* (Miranda Ribeiro)). The diagnosis of the genus was amended to include new features. The new species differ from each other mainly in terms of body shape, relative sucker size, and testes position. DNA sequences were obtained from *Creptotrema* spp. from Brazil, including 28S, ITS and COI. Genetic divergences among the new species and *C. creptotrema* varied from 2.1 to 5.2% (21–49 bp) for 28S, and 6.6 to 16.4% (21–45 bp) for COI. Phylogenetic analysis (28S) placed the newly generated DNA sequences of *Creptotrema* in a clade (*Creptotrema* clade *sensu stricto*) composed of *C. creptotrema*, the new species described herein, and all species previously described as *Auriculostoma*, revealing that *Auriculostoma* is best interpreted as a synonym of *Creptotrema* based on the principle of priority of zoological nomenclature. *Creptotrema funduli*, the single sequence of the genus previously available, was not grouped within the *Creptotrema* clade *sensu stricto*, suggesting the need for reevaluation of the taxonomic status of this species. Our results showed that *Creptotrema* represents a monophyletic genus of trematodes widely distributed across the Americas, which currently comprises 19 valid species of parasites of teleosts and anurans.

## Abstract (fr)

La diversité de *Creptotrema* a été étudiée à l’aide d’outils morphologiques et moléculaires, incluant des données pour *Creptotrema creptotrema* (espèce-type). Trois nouvelles espèces, parasites de poissons brésiliens, sont décrites : *Creptotrema conconae* n. sp. (hôte-type, *Imparfinis mirini* Haseman), *Creptotrema schubarti* n. sp. (hôte-type, *Characidium schubarti* Travassos) et *Creptotrema megacetabularis* n. sp. (hôte-type, *Auchenipterus osteomystax* (Miranda Ribeiro)). Le diagnostic du genre a été modifié pour inclure de nouvelles caractéristiques. Les nouvelles espèces diffèrent les unes des autres principalement en termes de forme du corps, de taille relative des ventouses et de position des testicules. Des séquences d’ADN ont été obtenues de *Creptotrema* spp. du Brésil, y compris 28S, ITS et COI. Les divergences génétiques entre les nouvelles espèces et *C. creptotrema* variaient de 2,1 à 5,2 % (21 à 49 pb) pour les séquences 28S, et de 6,6 à 16,4 % (21 à 45 pb) pour le COI. L’analyse phylogénétique (28S) a placé les séquences d’ADN nouvellement générées de *Creptotrema* dans un clade (*Creptotrema* clade sensu stricto) composé de *C. creptotrema*, les nouvelles espèces décrites ici et toutes les espèces précédemment décrites comme *Auriculostoma*, révélant qu’*Auriculostoma* est mieux interprété comme un synonyme de *Creptotrema* sur la base du principe de priorité de la nomenclature zoologique. *Creptotrema funduli*, la seule séquence du genre précédemment disponible, n’a pas été regroupée au sein du clade *Creptotrema* sensu stricto, ce qui suggère la nécessité d’une réévaluation du statut taxonomique de cette espèce. Nos résultats montrent que *Creptotrema* est un genre monophylétique de trématodes largement distribué à travers les Amériques, qui comprend actuellement 19 espèces valides, parasites de téléostéens et d’anoures.

## Introduction

Allocreadiidae Looss, 1902 includes relatively small digeneans that appear to be restricted to freshwater systems distributed across the Americas, Asia, and Europe [11, 24]. The cercariae develop in sphaeriid clams (Bivalvia) and encyst in aquatic arthropods (primarily insects and crustaceans). The adult forms inhabit the digestive system of teleosts and, occasionally, of reptiles (snakes), and amphibians (salamanders and frogs) [11].

The delimitation of genera in Allocreadiidae is usually defined by the possession of a combination of four non-unique morphological characters: the number of muscular “papillae” in the oral sucker; posterior extent of the intestinal ceca; posterior extent of the uterus, and anterior extent of the vitelline follicles. This approach has led to several controversies, resulting in polyphyletic groups and controversial genera and subgenera [11].

*Creptotrema* Travassos, Artigas & Pereira, 1928 was described to accommodate the type-species *Creptotrema creptotrema* Travassos, Artigas & Pereira, 1928 as allocreadiids presenting one pair of muscular ventrolateral papillae associated with the oral sucker and a uterus that does not reach the posterior region of the testes. Species of this genus parasitize freshwater teleosts belonging to the family Anostomidae in Brazil. There are currently seven valid species in this genus [26] (excluding *Creptotrema agonostomi* Salgado-Maldonado, Cabañas-Carranza & Caspeta-Mandujano, 1978, recently reassigned as *Pseudoparacreptotrema* Pérez Ponce de León, Pinacho-Pinacho, Mendoza-Garfias, Choudhury & García-Varela, 2016 [46]), described as parasites of teleosts in the United States, Brazil, Ecuador, Paraguay, and Argentina, and toads (Bufonidae) from Colombia.

Another “papillose genus” of allocreadiids of freshwater fishes from the Neotropical region is *Auriculostoma* Scholz, Aguirre-Macedo & Choudhury, 2004. This genus includes ten valid species of parasites of teleosts, most of which parasitize small-bodied characiforms from South America [25, 26]. According to Scholz et al. [58], the synapomorphy of *Auriculostoma*, relative to all other allocreadiid genera, is the presence of one pair of ventrolateral muscular papillae and one pair of prominent auricular dorsolateral papillae. However, recent studies [24, 53] using scanning electron microscopy (SEM) of specimens belonging to this genus refute the morphological description proposed by Scholz et al. [58], demonstrating the presence of a single pair of muscular lobes on either side of the oral sucker, thus raising questions about the classification of this group. Although molecular analyses, including the sequencing of *Auriculostoma* spp., have suggested the monophyly of the group, the lack of genome sequences of other “papillose genera” of allocreadiids from the Neotropical region has raised questions about the systematics of this trematode family.

As part of our long-term studies on the biodiversity of fish parasites from the tributaries of the Upper Paraná River basin in Brazil and streams from the Paranapanema River basin, three new species of *Creptotrema* are described, isolated from teleosts belonging to Siluriformes and Characiformes, supported by morphological and molecular data. For the first time, DNA sequences of *Creptotrema* spp. from Brazil were obtained, including the large subunit gene (28S rDNA), the internal transcribed spacer (ITS) region of ribosomal DNA, and the cytochrome c oxidase I gene (COI mtDNA) of mitochondrial DNA, including data for *C. creptotrema* from the type-locality and type-host.

The generic diagnosis of the genus was amended to accommodate the proposed new species and to reassign the species previously described as *Auriculostoma* (new combinations). The phylogenetic relationships among *Creptotrema* spp. and other digenean parasites of fishes were also evaluated, including the sequence of *Creptotrema funduli* Mueller, 1934 from North America, the single previously sequenced species also considered a member of *Creptotrema*.

## Materials and methods

### Ethics

All applicable institutional, national, and international guidelines for the ethical handling of animals and for collection of zoological material were followed (SISBio #60102-1; SISBio #577/2015; SISBio #37910-4) including recommendations from the Ethics Committee for Animal Experimentation (CEUA-UNESP #942; CEUA-UNESP #120; CEUA-UEL #3014.2018.86). According to Brazilian laws, species registration for scientific research purposes was carried out at SisGen (AD2D499).

### Host sampling and parasitological procedures

Fifty specimens of *Imparfinis mirini* Haseman, 1911, 39 specimens of *Cetopsorhamdia iheringi* Schubart & Gomes, 1959 (23° 9′ 40.8′′ S, 48° 53′ 7.3′′ W), and 60 specimens of *Characidium schubarti* Travassos, 1955 (23° 9′ 25.7′′ S, 48° 48′ 31.3′′ W) were collected from streams of the Middle Paranapanema River, in the Upper Paraná River basin, Avaré municipality, São Paulo State, Brazil. Fishes were captured between August 2016 and September 2017, using a seine net with a small mesh (mosquito net fishing), cast nets, and a hand net. A total of 32 specimens of *Auchenipterus osteomystax* (Miranda Ribeiro, 1918) were collected from the Aguapeí River, a tributary of the Paraná River, in Castilho municipality (21° 03′ 03.16′′ S, 51° 45′ 58.16′′ W), São Paulo State, between August 2013 and January 2014, using nylon monofilament gill nets and cast nets. One specimen of *Megaleporinus obtusidens* (Valenciennes, 1837) (= *Leporinus elongatus*) was collected in Cachoeira de Emas (Emas Waterfalls) (21º 55′ 26.4′′ S, 47º 21′ 37.3′′ W), Mogi-Guaçu River, Pirassununga municipality, São Paulo State, Brazil, in October 2019, using a hook-and-line fishing method.

Voucher specimens of the fish hosts were fixed in 10% formalin, preserved in 70% ethanol, and deposited in Brazilian fish collections: Laboratory of Fish Biology and Genetics of the São Paulo State University (UNESP), São Paulo State (*I. mirini*: LBP 29051; *C. iheringi*: LBP 29050; *C. schubarti*: LBP 29045), and the Zoology Museum of the State University of Londrina (UEL) (*M. obtusidens*: MZUEL 20604). The scientific names of the hosts follow those of Fricke et al. [22].

All fishes were individually euthanized, stored in plastic bags, and placed in a styrofoam box with ice for transportation to the laboratory for necropsy. The intestines of the fishes were removed and analyzed using a stereomicroscope. Some of the collected specimens of digeneans were preserved directly in 96% ethanol for molecular analyses; others were placed in hot water (~60 °C) and subsequently stored in 70% ethanol for morphological procedures or fixed directly in hot 70% ethanol for SEM preparations.

For analyses of the internal organs, the digenean specimens were stained with chloride carmine, Mayer’s carmalumen, or Gomori’s trichrome, cleared in eugenol, and mounted on permanent slides using Canada balsam. Some specimens were mounted in Hoyer’s or Gray and Wess’ medium to highlight the visualization of the eggs.

Morphometrical and morphological analyses of these specimens were performed using a computerized image analysis system with differential interference contrast (DIC) (Leica Application Suite, V3; Leica Microsystems, Wetzlar, Germany). The morphological descriptions followed the recommendations of Travassos et al. [68], Kohn [29], and observations by Scholz et al. [58] and Razo-Mendivil et al. [53]. All measurements are presented in micrometers (μm) and are expressed as means, followed by the range in parentheses. Illustrations of the structures were produced with the aid of a camera lucida mounted on a Leica DMLS microscope with phase-contrast optics. The prevalence and mean abundance of infections were calculated according to Bush et al. [10].

The SEM analyses were conducted on the specimens of *C. creptotrema* at the Laboratório de Microscopia Eletrônica e de Microanálise (LMEM) of the State University of Londrina (UEL), in the municipality of Londrina, Paraná State, Brazil. The specimens were processed through an increasing series of alcoholic dehydration (70%–100%) (adapted from [2]). Subsequently, the specimens were dried at the critical point with carbon dioxide, metalized in gold, and photographed using a Quanta 200 SEM-FEI Company microscope. The final images were edited using CorelDRAW X6 software (Corel Corporation, Canada).

Holotype and paratypes of the new species of *Creptotrema* were deposited in the Helminthological Collection of the Oswaldo Cruz Institute (CHIOC), Rio de Janeiro State, Brazil. Other vouchers were deposited in the Helminthological Collection of the Department of Biostatistics, Plant Biology, Parasitology and Zoology, Institute of Biosciences, São Paulo State University, UNESP (CHIBB), Botucatu municipality, São Paulo State, Brazil.

Vouchers of *Creptotrema* species were examined for comparative purposes [*C. creptotrema* (CHIOC 32110, 32187, 35584, 36573, 36995, 5970, 6086–89); *Creptotrema paraense* Vicente, Santos & Souza, 1978 (= *Creptotrema paraensis*) (CHIOC: 20970, 31205); *Creptotrema lynchi* Brooks, 1976 (CHIOC 31647–31648); *Auriculostoma platense* (Szidat, 1954) Scholz, Aguirre-Macedo & Choudhury, 2004 (= *Crepidostomum platense*) (CHIOC 32188); *Creptotrematina dissimilis* (Freitas, 1941) Yamaguti, 1954 (= *Creptotrema dissimilis*) (CHIOC 10719, 11438, 12669); and *Creptotrematina dispar* (Freitas, 1941) Manter, 1962 (*= Creptotrema dispar*) (CHIOC 15357)]. Photomicrographs of the vouchers of *C. funduli* (CMNPA1989–0566) deposited in the Canadian Museum of Nature, *C. lynchi* (UNSM20251) deposited at the University of Nebraska State Museum, and *Auriculostoma guacurarii* Montes, Barneche, Croci, Balcazar, Almirón, Martorelli & Pérez-Ponce de León, 2021 deposited in the Museo de Ciencias Naturales de La Plata (MLP-He 7713, MLP-He 7714) were also examined.

### DNA extraction, amplification, sequencing, and alignments

To confirm morphological identification, each parasite specimen was mounted on a slide with 96% ethanol, covered with a coverslip, and photographed. Conspecific specimen vouchers (paragenophores, according to Pleijel et al. [50]) were mounted in Canada balsam medium and deposited in CHIOC (*C. creptotrema* [CHIOC 39702]; *C. conconae* n. sp. [CHIOC 39596 a–g]; *C. schubarti* n. sp. [CHIOC 39599] and *C. megacetabularis* n. sp. [CHIOC 39703]).

Genomic DNA was extracted from the species described herein (three specimens of *C. creptotrema*, and specimens of the new species described herein: one specimen of *Creptotrema conconae* n. sp., one specimen of *Creptotrema schubarti* n. sp., and four specimens of *Creptotrema megacetabularis* n. sp.), using the DNeasy Blood & Tissue Kit (Qiagen, Valencia, CA, USA), following the manufacturer’s protocol. Fragments of 28S rDNA, ITS rDNA, and COI mtDNA were amplified using the primers and cycling conditions presented in Table 1. Conventional polymerase chain reaction (PCR) amplifications were performed on a final volume of 25 μL using ready-to-go PCR beads (GE Healthcare) with extracted DNA (3.0 μL) and 1.0 μL of each PCR primer. PCR products (2.0 μL) were run on an agarose gel (1%) using GelRed™ fluorescent nucleic acid dye and loading buffer to confirm amplicon size and yield. PCR amplicons were purified using the QIAquick PCR Purification Kit (Qiagen), following the manufacturer’s instructions. Automated sequencing was performed directly on purified PCR products using a BigDye v.3.1 Terminator Cycle Sequencing Ready Reaction kit on an ABI 3500 DNA genetic sequencer (Applied Biosystems). The new sequences were assembled and edited using Sequencher v. 5.2.4 (Gene Codes, Ann Arbor, MI, USA).

**Table 1:** Primers used in this study.

Gene/region	Primers and sequences 5′–3′	Cycling conditions	References
28S	LSU	Initial denaturation of 7 min at 95 °C; 40 cycles of 40 s at 95 °C, 45 s at 57 °C and 1 min and 30 s at 72 °C; 10 min at 72 °C for final extension	[34, 43]
	TAGGTCGACCCGCTGAAYTTAAGCA		
	1500R		
	GCTATCCTGAGGGAAACTTCG		
	1200R		
	GCATAGTTCACCATCTTTCGG		
ITS	GLYP1	Initial denaturation of 7 min at 95 °C; 40 cycles of 40 s at 95 °C, 45 s at 57 °C and 1 min 30 s at 72 °C; 10 min at 72 °C for final extension	[36, 56]
	GCTGAGAAGACGACCAAACTTGAT		
	BD2		
	TATGCTTAAATTCAGCGGGT		
COI	JB3	Initial denaturation of 5 min at 94 °C; 40 cycles of 30 s at 92 °C, 45 s at 47 °C and 1 min 30 s at 72 °C; 10 min at 72 °C for final extension	[42, 55]
	TTTTTTGGGCATCCTGAGGTTTAT		
	JB4.5		
	TAAAGAAAGAACATAATG AAAATG		

The obtained ITS rDNA sequences were deposited in the GenBank database. However, no phylogenetic analysis was conducted because of the shortage of sequences of this marker from allocreadiids deposited in the databank, culminating in an alignment with a reduced number of base pairs. The phylogenetic analyses presented herein were performed using sequences of 28S rDNA and COI mtDNA. Two independent datasets were created: the first contained the newly generated 28S rDNA sequences of *C. creptotrema* and three new *Creptotrema* spp., along with 79 published sequences of Allocreadiidae retrieved from GenBank and species from the genera *Prosthenhystera* Travassos, 1922 (Callodistomidae), *Dicrocoelium* Dujardin, 1845 (Dicrocoeliidae), *Degeneria* Campbell, 1977, and *Phyllodistomum* Braun, 1899 (both Gorgoderidae), used as the outgroup. The second contained the newly generated COI mtDNA sequences of *C. creptotrema* and the three new *Creptotrema* spp., along with 16 published sequences of the Allocreadiidae retrieved from GenBank and *Phyllodistomum parasiluri* Yamaguti, 1934, and *Dicrocoelium* spp. as the outgroup (Table 2).

**Table 2:** List of digeneans included in the phylogenetic analyses, with details of the host, locality and GenBank accession numbers of sequences from the 28S rDNA and mitochondrial cytochrome c oxidase I (COI mtDNA) genes. New sequences obtained for the present study are in bold.

Digeneans	Host	Host order	Locality	GenBank ID			Reference
				28S rDNA	COI mtDNA		
Allocreadiidae							
*Acrolichanus auriculatus*	*Acipenser schrenckii*; *Acipenser ruthenus*; *Huso dauricus*	Acipenseriformes	Russia	MN524579–MN524580; MN524583; MN524583; FR821397–FR821398		–	[3, 5]
*Allocreadium gotoi*	*Misgurnus anguillicaudatus*	Cypriniformes	Iiyama City, Japan	LC215274		–	[59]
*Allocreadium isoporum*	*Alburnus alburnus*	Cypriniformes	Oster Lake, Karelia	GU462125–GU462126		–	[47]
*Allocreadium lobatum*	*Semotilus atromaculatus*	Cypriniformes	Tobacco Creek, Canada	–		KC899847	[39]
*Allocreadium neotenicum*	*Hydroporus rufifrons*	Coleoptera*	Cumbria, England	JX977132		–	[8]
*Bunodera luciopercae*	*Gymnocephalus cernuus*/*Perca fluviatilis*	Perciformes	Tvertsa River, Russia	GU462122/GU462123		–	[47]
*Bunodera vytautasi*	*Pungitius pungitius*	Gasterosteiformes	Magadan region, Chernoe Lake, Russia	MG262545		–	[4]
*Bunodera mediovitellata*	*Gasterosteus aculeatus*	Gasterosteiformes	Kamchatka, Azabachye River, Russia	MG262549		–	[4]
*Crepidostomum affine*	*Hiodon tergisus*	Hiodontiformes	Pearl River, Mississippi, USA	KF356363		–	[66]
*Crepidostomum auritum*	*Aplodinotus grunniens*	Perciformes	Pearl River, Mississippi, USA	KF356373		–	[66]
*Crepidostomum cornutum*	*Lepomis gulosus*	Perciformes	Pascagoula River, Mississippi, USA	KF356374		–	[66]
*Crepidostomum farionis*	*Oncorhynchus masou/ Pisidium casertanum*	Salmoniformes/ Sphaeriida*	Russia/Takvatn Lake, North of Norway	FR821403–FR821404/KY513138		–	[3, 60]
*Crepidostomum illinoiense*	*Hiodon alosoides*	Hiodontiformes	Missouri River, North Dakota, USA	KF356372		–	[66]
*Crepidostomum metoecus* (= *Crepidostomum nemachilus*)	*Salvelinus leucomaensis*; *Barbatula toni*; *Gammarus lacustris*	Salmoniformes; Cypriniformes; Amphipoda*	Russia/Lake Takvatn, Northern Norway	FR821406–FR821407; FR821408–FR821409; KY513145		–	[3, 60]
*Crepidostomum oschmarini*	*Pisidium casertanum*	Sphaeriida*	River Nedzingė, Lithuania	MH159994		–	[49]
*Creptotrema astyanace* comb. n. (= *Auriculostoma astyanace*)	*Astyanax aeneus*/ *Astyanax fasciatus*	Characiformes	Guanacaste, Costa Rica	HQ833707/KF631422		–	[17, 53]
***Creptotrema creptotrema***	*Megaleporinus elongatus* (= *Leporinus elongatus*)	Characiformes	Mogi-Guaçu River, Upper Paraná River basin, Brazil	**OK044371** **–** **OK044372**		**OK075290** **–** **OK075292**	**Present study**
***Creptotrema conconae* n. sp.**	*Imparfinis mirini*	Siluriformes	Upper Paraná River basin, Brazil	**OK044374**		**OK075288**	**Present study**
*Creptotrema funduli* (*species inquirenda*)	*Fundulus notatus*	Cyprinodontiformes	Biloxi River, Mississippi, USA	JQ425256		–	[15]
*Creptotrema guacurarii* comb. n. (= *Auriculostoma guacurarii*)	*Characidium heirmostigmata*	Characiformes	Iguazú National Park, Arrechea Stream, Argentina	MN822004		–	[41]
*Creptotrema lobata* comb. n. (= *Auriculostoma lobata*)	*Brycon guatemalensis*	Characiformes	El Managal Lagoon, Tenosique, Mexico	KX954170–KX954173		–	[24]
***Creptotrema megacetabularis* n. sp.**	*Auchenipterus osteomystax*	Siluriformes	Upper Paraná River basin, Brazil	**OK044375** **–** **OK044378**		**OK075289**	**Present study**
***Creptotrema schubarti* n. sp.**	*Characidium schubarti*	Characiformes	Upper Paraná River basin, Brazil	**OK044373**		**OK075293**	**Present study**
*Creptotrema* cf. *stenopteri* comb. n. (= *Auriculostoma* cf. *stenopteri*)	*Charax stenopterus*	Characiformes	La Plata River, Argentina	MN822005		–	[41]
*Creptotrema tica* comb. n. (= *Auriculostoma tica*)	*Gymnotus maculosus*	Gymnotiformes	Orosí River, Costa Rica	MH997001–MH997002		–	[25]
*Creptotrema totonacapanense* comb. n. (= *Auriculostoma totonacapanensis*)	*Astyanax mexicanus*	Characiformes	Filipinas, Veracruz, Mexico	KF631417–KF631420		–	[53]
*Creptotrematina aguirrepequenoi*	*Astyanax aeneus*/ *Astyanax mexicanus*	Characiformes	Tempisquito River, Guanacaste, Costa Rica/ Filipinas creek, Veracuz, Mexico	HQ833709/KF631421		–	[17, 53]
*Creptotrematina batalhensis*	*Astyanax lacustris*	Characiformes	Batalha River, Brazil	MT512642		–	[19]
*Margotrema bravoae*	Goodeidae; *Allotoca diazi*; *Zoogoneticus quitzeoensis*; *Allotoca meeki*	Cyprinodontiformes	Mexico (Central region)	KT833278		KC899900; KC899906; KC899908; KC899922; KC899925; KC899934; KC899937; KC899952	[39, 45]
*Margotrema resolanae*	Goodeidae; *Xenotaenia resolanae*	Cyprinodontiformes	Mexico (Central region)	KT833272		KC899859; KC899861–KC899863	[39, 45]
*Megalogonia ictaluri*	*Ictalurus punctatus*	Siluriformes	Pearl River, Mississipi, USA	EF032694		–	[16]
*Paracreptotrema blancoi*	*Priapichthys annectens*	Cyprinodontiformes	Orosí River, Costa Rica	KT833283/KT833285		–	[45]
*Paracreptotrema rosenthali*	*Xiphophorus malinche*	Cyprinodontiformes	Malila River, Hidalgo, Mexico	KT833287–KT833288		–	[45]
*Paracreptotrematoides heterandriae* (=*Paracreptotrema heterandriae*)	*Heterandria bimaculata*	Cyprinodontiformes	Agua Bendita, Xico, Veracruz, Mexico	KF697696–KF697697		–	[45, 53]
*Pseudoparacreptotrema axtlaense* (=*Pseudoparacreptotrema axtlaensis*	*Dajaus monticola*	Perciformes	Río Axtla, Axtla de Terrazas, San Luis Potosí, Mexico	MT180831–MT1808332		–	[46]
*Pseudoparacreptotrema falciformis*	*Dajaus monticola*	Perciformes	River at Matías Romero, Oaxaca, Mexico	MT180824–MT180830		–	[46]
*Pseudoparacreptotrema macroacetabulatum* (=*Pseudoparacreptotrema macroacetabulata*)	*Profundulus guatemalensis*	Cyprinodontiformes	Puente Sansare, Guatemala	KT833320		–	[45]
*Pseudoparacreptotrema pacificum*	*Dajaus monticola*	Perciformes	Puente Novillero, Chiapas, Mexico	MT180810–MT180811; MT180818–MT180819; MT180822–MT180823		–	[46]
*Pseudoparacreptotrema profundulusi*	*Profundulus* sp.	Cyprinodontiformes	Templo River, San Juan del Río, Oaxaca, Mexico	KT833290		–	[45]
*Wallinia brasiliensis*	*Astyanax fasciatus*	Characiformes	Upper Paraná River Basin, Brazil	MH520995		–	[20]
*Wallinia anindoi*	*Astyanax aeneus*	Characiformes	San Juan, Oaxaca, Mexico; San Juan River, Chiapas, Mexico; las Cabezas River, El Progreso, Guatemala	MH997003–MH997004		–	[25]
*Wallinia caririensis*	*Astyanax bimaculatus*	Characiformes	Batateiras River, Brazil	MW024866; MW024899		–	[18]
*Wallinia chavarriae*	*Astyanax aeneus/ Gephyrocharax* sp.	Characiformes	Animas River, Guanacaste, Costa Rica/ Gamboa, Panama	HQ833703		KC899851–KC899853	[17, 39]
*Wallinia mexicana*	*Astyanax mexicanus*	Characiformes	Covadonga River, Durango, Mexico; Huichihuayan River, San Luis Potosí, Mexico	KJ535504–KJ535505		–	[44]
Gorgoderidae							
Degeneria halosauri**	*Halosauropsis macrochir*	Notacanthiformes	Atlantic Ocean	AY222257		–	[43]
Phyllodistomum angulatum**	*Sander lucioperca*	Perciformes	Chesnava River, Russia	KJ729531		–	[48]
Phyllodistomum folium**	*Gymnocephalus cernua*	Perciformes	Curonian Lagoon, Lithuania	KX957729		–	[62]
Phyllodistomum macrocotyle**	*Dreissena polymorpha*	Veneroida	Lake Lepelskoe, Belarus	AY288828		–	[63]
Phyllodistomum parasiluri**	*Silurus asotus*	Siluriformes	Takashima City, Japan	–		LC002524	[69]
Dicrocoeliidae							
Dicrocoelium chinensis**	*Bos grunniens*	Artiodactyla	Maqu (Gansu Province), China	–		NC025279	[33]
Dicrocoelium dendriticum**	*Marmota bobak*	Rodentia	Kharkiv region, Ukraine	AF151939		NC025280	[33, 67]
Callodistomidae							
Prosthenhystera caballeroi**	*Astyanax aeneus*	Characiformes	Tampisquto River, Guanacaste, Costa Rica	KM871186		–	[65]
Prosthenhystera obesa**	*Hoplias* sp.	Characiformes	Itaya River, Iquitos, Peru	EF032690		–	[43]
Prosthenhystera oonastica**	*Pylodictis olivaris*	Siluriformes	Pearl River, Mississippi, USA	KM871182		–	[65]

* Invertebrate hosts that harbor the larval forms of the parasites.

** Species used as outgroup.

The alignments were performed using the MUSCLE algorithm implemented on Geneious 7.1.3 [27] with default settings. The presence of stop codons and indels for the COI mtDNA alignment was verified by amino acid translation using the trematode mitochondrial code table on Geneious 7.1.3 [27]. To evaluate the occurrence of substitution saturation, the I_ss_ indices were estimated using DAMBE 5 software [72].

### Phylogenetic analyses

The best-fitting models for nucleotide substitution selected by jModelTest software [51] using the Akaike information criterion were GTR + I + G for the 28S rDNA and HKY + G for the COI mtDNA datasets. Phylogenies were reconstructed for each alignment under Bayesian inference (BI) using MrBayes v. 3.2 and maximum-likelihood (ML) on RAxML v. 8 [61], both implemented on the CIPRES web portal [40]. The BI was run using four Markov chain Monte Carlo searches with 10,000,000 generations and sampling tree topologies every 100 generations. The burning was set to the first 25% of generations; the consensus trees were estimated using the remaining topologies. The nodes with posterior probabilities greater than 0.90 were considered well supported. The ML analyses were estimated using random starting trees and 1000 bootstrap replicates; nodes with bootstrap values greater than 70 were considered to be well supported. The final trees were visualized and edited using FigTree v. 1.3.1 [52] and CorelDRAW X6.

Pairwise genetic distances within and among sequences were calculated using the Kimura-2-parameter (K2P) model and a bootstrap procedure with 1000 replicates in the MEGA7 program [28, 32].

## Results

### *Diagnosis of the genus* Creptotrema

Allocreadiidae Looss, 1902

*Creptotrema* Travassos, Artigas & Pereira, 1928

**Type species, host, and locality:** *Creptotrema creptotrema* Travassos, Artigas & Pereira, 1928 from *Megaleporinus obtusidens* (Valenciennes, 1837) (= *Leporinus elongatus*)*,* Cachoeira de Emas (Emas Waterfalls), Pirassununga municipality, São Paulo State, Brazil*.*

**Other species:** *Creptotrema funduli* Mueller, 1934 (*species inquirenda*); *Creptotrema macrorchis* (Szidat, 1954) comb. n.; *Creptotrema platense* (Szidat, 1954) comb. n.; *Creptotrema stenopteri* (Mañé-Garzón & Gascón, 1973) comb. n.; *Creptotrema lynchi* Brooks, 1976; *Creptotrema paraense* Vicente, Santos & Souza, 1978; *Creptotrema pati* Lunaschi, 1985; *Creptotrema astyanace* (Scholz, Aguirre-Macedo & Choudhury, 2004) comb. n.; *Creptotrema lamothei* Curran, 2008; *Creptotrema sucumbiosa* Curran, 2008; *Creptotrema diagonale* (Curran, Tkach & Overstreet, 2011) comb. n.; *Creptotrema foliaceum* (Curran, Tkach & Overstreet, 2011) comb. n.; *Creptotrema totonacapanense* (Razo-Mendivil, Mendoza-Garfias, Pérez-Ponce de Léon & Rubio-Godoy, 2014) comb. n.; *Creptotrema lobata* (Hernández-Mena, Lynggaard, Mendoza-Garfias & Pérez-Ponce de León, 2016) comb. n.; *Creptotrema tica* (Hernández Mena, Pinacho-Pinacho, García-Varela, Mendoza-Garfias & Pérez-Ponce de León, 2018) comb. n.; *Creptotrema guacurarii* comb. n. (Montes, Barneche, Croci, Balcazar, Almirón, Martorelli & Pérez-Ponce de León, 2021); *Creptotrema conconae* n. sp. (present study); *Creptotrema schubarti* n. sp. (present study); *Creptotrema megacetabularis* n. sp. (present study).

**Amended diagnosis:** Body small, round to elongate or lanceolate, dorsoventrally flattened. Slight constriction at prepharyngeal level (neck region) present or absent. Tegument smooth. Remnants of eyespots present (most evident in young specimens, adults in the early development phase) or absent. Oral sucker subterminal, rounded or funnel-shaped with variable morphology (a ventral anterior horseshoe-shaped structure and presence of a discrete single pair of muscular lobes [“auricles”] on either side of oral sucker, stretching from the ventral side to lateral area, but not extending to dorsal region, or a single pair of muscular lobes on either side of oral sucker, with a broad base, stretching from ventrolateral to dorsolateral side forming an auricular dorsolateral papilla, with a free end). Small dome-like papillae over the margin of oral sucker (around 19–21) and ventral sucker (around 6), with a variable arrangement. Ventral sucker round, pre-equatorial to equatorial, with similar size or larger than oral sucker. Prepharynx short or absent. Pharynx subspherical. Esophagus short to moderately long. Intestinal ceca blind, bifurcating at anterior margin of ventral sucker; ceca end immediately or well posteriorly beyond testes. Genital pore median, at level of intestinal bifurcation or slightly anterior or posterior, equidistant between ventral sucker and cecal bifurcation. Seminal vesicle bipartite; pars prostatica and cirrus present. Cirrus unarmed, eversible. Cirrus-sac well-developed, long, usually forming several loops, sometimes overlapping ventral sucker or reaching the ovary posteriorly. Testes in posterior half of body, oval or elongate, entire or slightly lobed, tandem, symmetrical or oblique, intra or extracecal. Ovary posterolateral to ventral sucker, slightly pre-equatorial to equatorial, pretesticular, entire to lobed, submedian. Seminal receptacle posterior to ovary. Laurer’s canal with dorsolateral opening. Vitelline follicles lateral, exclusively extracecal or extra- and intracecal, with large or small follicles extending from level of oral sucker to posterior end of body, completely separated into two fields or confluent (scarcely or not) in post-testicular region. Uterus pretesticular, between anterior testis and genital pore, but loops may extend to the post-testicular region; intra- or extracecal. Eggs operculate, unembryonated, without spines or filaments; small knob opposite to operculum present or absent. Excretory pore terminal at posterior region of body; excretory vesicle I-shaped to claviform, reaching to anterior testis. Intestinal parasites of freshwater fishes and anurans in the Americas.

**Remarks:** Travassos et al. [68] originally described the defining characters of *Creptotrema* as the presence of one pair of muscular ventrolateral lobes (= “papillae” in original description) associated with the oral sucker, and a uterus that does not reach the region posterior to the testes. In the present study, the generic diagnosis was amended, adding new features to accommodate the three new species proposed in the present study, and also amending the reassignment of the species previously described as belonging to *Auriculostoma* (new combinations).

*Auriculostoma* was described as a new genus of parasites of freshwater fishes, accommodating *Auriculostoma astyanace* Scholz, Aguirre-Macedo & Choudhury, 2004 (type-species), a parasite of the characid *Astyanax fasciatus* (Cuvier, 1819) from Nicaragua [58]. Further comparison with other allocreadiids from South America revealed that the morphology of *A. astyanace* is similar to the other three species originally described as belonging to *Crepidostomum* Braun, 1900. Thus, these species were included in the genus as *Auriculostoma platense* (Szidat, 1954) (= *Crepidostomum platense*), *Auriculostoma macrorchis* (Szidat, 1954) (= *Crepidostomum macrorchis*), and *Auriculostoma stenopteri* (Mañé-Garzón & Gascón, 1973) (= *Crepidostomum stenopteri*) [58].

According to Scholz et al. [58], the synapomorphy of *Auriculostoma* to all other allocreadiid genera is the presence of two pairs of muscular lobes (originally described as “papillae”), one ventrolateral and one pair prominent, auricular dorsolateral. However, Razo-Mendivil et al. [53], using SEM photomicrographs of specimens of *Auriculostoma totonacapanense* Razo-Mendivil, Mendoza-Garfias, Pérez-Ponce de Léon & Rubio-Godoy, 2014, demonstrated the presence of a single narrow muscular lobe on either side of the oral sucker, with a broad base, stretching from the ventrolateral to the dorsolateral side, and free ends (see Fig. 2 of [53]). The same morphology was confirmed using SEM analysis for *Auriculostoma lobata* [24] and *Auriculostoma tica* [25]. SEM analysis also revealed the presence of small dome-like papillae over the margins of oral and ventral suckers that were poorly visualized by light microscopy. In the present study, we adopted the term “lobes” to refer to prominent and muscular structures (“auricles”) present in the superior margin of the oral sucker, while the term “papillae” was used to refer to these small dome-like structures over the oral and ventral suckers.

**Figure 1:**
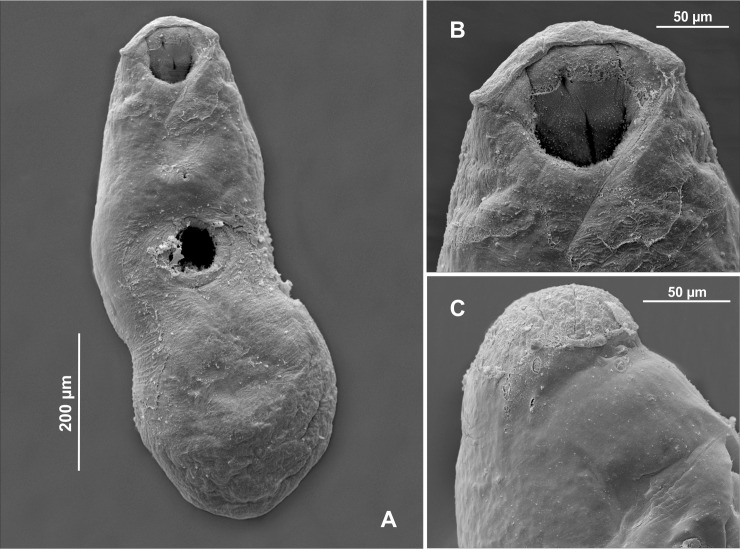
Scanning electron photomicrographs of a specimen of *Creptotrema creptotrema* Travassos, Artigas & Pereira, 1928 (A) Total view; (B) Ventral view of the oral sucker with an anterior horseshoe-shaped structure and presence of a discrete single ventrolateral muscular lobe on either side of the oral sucker; (C) Lateral view of the discrete lobe that stretches from the ventral side to lateral area, not extending to the dorsal side.

Following the analyses of the species originally described as *Creptotrema* and *Auriculostoma* deposited in museums, it was possible to observe the same morphological pattern of the oral sucker described by Razo-Mendivil et al. [53] for the majority of species that varied in the presence or absence of dorsal free ends. The same morphological pattern was observed in the SEM analyses of the newly collected specimens of *C. creptotrema* in the present study (Figs. 1A–1C), *in vivo* specimens (of the new species described and *C. creptotrema* analyzed herein), and the illustrations provided in the descriptions of new species over the years.

Therefore, the main amendments to the original diagnosis of the genus *Creptotrema* are the presence of remnants of eyespots in some species (numerous and evident in young and adults in the early development phase, *versus* not present in the original description by Travassos et al. [68]); the presence of ovary and testes with entire or slightly lobed margins, *versus* simply entire in the original description of *Creptotrema*; the presence of small dome-like papillae over the margin of the oral sucker and ventral sucker, with variable arrangements (more visible by SEM analysis); and the presence of an oral sucker subterminal, rounded or funnel-shaped, with variable morphology of the lobes. The oral sucker may form a discrete lobe on either side of the oral sucker, without prominent dorsolateral lobes (“auricles”) and free ends on the dorsal view (as originally described for the type-species *C. creptotrema* and other congeners such as *C. lynchi* and *C. megacetabularis* n. sp.); or assuming the same morphology described by Razo-Mendivil et al. [53] and observed for most species previously attributed to *Auriculostoma* and some species of *Creptotrema* (*C. paraense*, and the new species described herein as *C. conconae* n. sp.).

Although the uterus was originally described as pretesticular, it may extend to the inter-testicular (as observed in *C. guacurarii* comb. n.) or post-testicular regions, depending on the number of eggs present in the uterus and the maturity of the specimens (as amended for *C. creptotrema* in this study, and described for *C. schubarti* n. sp. and *C. megacetabularis* n. sp.); it may occupy the extracecal region (as described for *C. lynchi* and *C. megacetabularis* n. sp.) or the intra-cecal region (as in other congeners).

The morphological and molecular evidence presented in this study supports the recognition of *Auriculostoma* as a synonym of *Creptotrema*.

### *Creptotrema creptotrema* Travassos, Artigas & Pereira, 1928 (Figs. 1A–1C; Figs. 2A–2D; Figs. 3A–3D)

Synonym: *Creptotrema lynchi* [30, 71]

**Figure 2:**
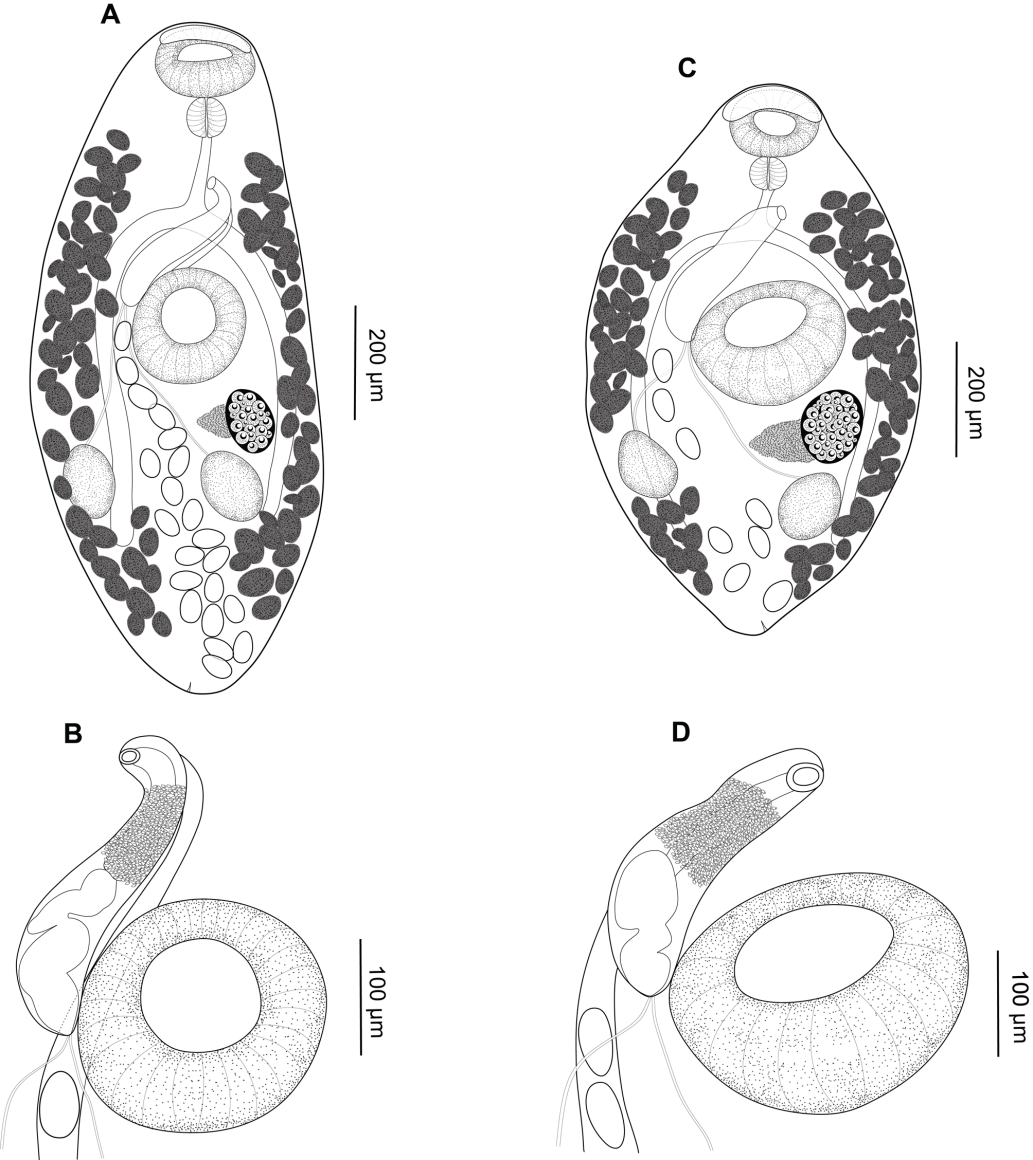
*Creptotrema creptotrema* Travassos, Artigas & Pereira, 1928 from *Megaleporinus obtusidens* (= *Leporinus elongatus*) (Characiformes, Anostomidae) collected in Cachoeira de Emas (Emas Waterfalls) for the present study: (A) Completely developed adult; (B) Detail of the cirrus-sac; (C) Adult in the early development phase; (D) Detail of the cirrus-sac.

**Figure 3:**
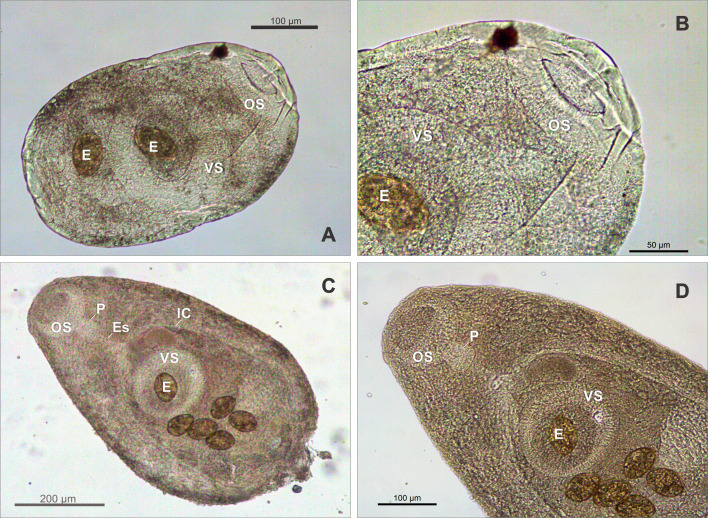
Specimens of *Creptotrema creptotrema* Travassos, Artigas & Pereira, 1928 sequenced in the present study: (A–B) Adult in the early development phase (GenBank accession numbers: OK044371; OK075290); (C–D) Completely developed adult (GenBank accession numbers: OK044372; OK075291). OS: oral sucker; VS: ventral sucker; E: egg; P: pharynx; Es: Esophagus; IC: intestinal ceca.

*Type-host*: *Megaleporinus obtusidens* (Valenciennes, 1837) (= *Leporinus elongatus*) (Characiformes, Anostomidae) [23, 67].

*Other hosts: Pimelodus maculatus* Lacepède, 1803 (Siluriformes, Pimelodidae) [31], *Conorhynchos conirostris* (Valenciennes, 1840) (Siluriformes, Pimelodidae) [7], and *Tetragonopterus chalceus* Spix & Agassiz, 1829 (Characiformes, Characidae) [1].

*Type-locality:* Emas Waterfalls (Cachoeira de Emas) (21° 55′ 26.4′′ S, 47° 21′ 37.3′′ W), Mogi-Guaçu River, Pirassununga municipality, São Paulo State, Brazil [68].

*Other localities:* Middle Paraná River, Province of Corrientes, Argentina [23]; Guaíba Estuary, Rio Grande do Sul State, Brazil [31, 71]; Três Marias reservoir, Upper São Francisco River, Minas Gerais State, Brazil [1, 7].

*Site of infection:* intestine.

*Prevalence and mean abundance (present study)*: one host analyzed and infected with 17 specimens.

*Material deposited (present study):* vouchers CHIOC 39702 a–g.

*Representative DNA sequence (present study):* 1188 and 1190 bp long sequences of 28S rDNA gene – GenBank OK044371 and OK044372; 426, 447, and 447 bp long sequences of COI mtDNA – GenBank OK075290, OK075291 and OK075292.

**Redescription**: (Based on specimens collected for the present study: 5 completely developed adults, stained with chloride carmine). Body small, round to elongated, 1248 (1120–1405) long, 564 (513–663) wide. Tegument smooth. Oral sucker subterminal, rounded to funnel-shaped, 163 (141–206) long, 188 (175–194) wide, with ventral anterior horseshoe-shaped flaps, and a discrete single ventrolateral muscular lobe (“auricles”) on either side of oral sucker, stretching from ventral region to lateral area, but not extending to dorsal region. Ventral sucker round, equatorial, 249 (232–278) long, 257 (223–305) wide. Ratio of oral sucker length to ventral sucker length 1:1.6 (1.3–1.7); ratio of oral sucker width to ventral sucker width 1:1.4 (1.2–1.6). Prepharynx short. Pharynx subspherical 73 (67–82) long, 81 (68–93) wide. Esophagus 142 (87–216) long. Intestinal ceca extending to posterior end of body. Genital pore median, at level of pharyngeal zone or intestinal bifurcation. Cirrus-sac well-developed, 316 (258–420) long, exceeding length of ventral sucker, with basal seminal vesicle, pars prostatica occupying most of anterior end of cirrus-sac, and unarmed and eversible cirrus. Testes in posterior half of body, 151 (125–184) long, 107 (89–143) wide, oval to elongated, entire margins, symmetrical to oblique. Ovary 160 (151–181) long, 147 (119–182) wide, posterolateral to ventral sucker, pretesticular, entire margins. Vitelline follicles large, extending from level of oral sucker to posterior end of body; follicles predominantly extracecal and cecal, completely separated into two lateral fields. Mehlis’ gland close to ovary, triangular, dextral to ovary. Laurer’s canal not observed. Uterus intra-cecal, extending between testes and genital pore in young specimens (adults in the early development phase) or may exceed testes and extend to posterior region of body in completely developed adults. Eggs operculate, large, 66 (55–80) long, 36 (31–43) wide. Excretory pore terminal at posterior region of body; excretory vesicle I-shaped, reaching testicular region.

**Remarks:** The original description of *C. creptotrema* [68] was revised by Kohn [29] to resolve inconsistencies about morphological features such as the absence of spines in the tegument (originally described as spinous), and to provide a consistent description of the cirrus sac (absent in the original description). The redescription provided by Kohn [29] was made based on the same specimens analyzed by Travassos et al. [68], deposited at the Helminthological Collection of the Oswaldo Cruz Institute (CHIOC).

In the present study, we collected new specimens of *C. creptotrema* obtained from the type-host *M. obtusidens* (Anostomidae) and type-locality (Emas Waterfalls) in Brazil and compared them with all the materials previously deposited in the CHIOC by Travassos et al. [68]. The specimens from the present study were obtained from a single host and presented morphological variations in body size and shape, the ratio between oral and ventral sucker, and the number of eggs in the uterus.

Some specimens were similar to those described by Travassos et al. [68] (Figs. 2C–2D; Figs. 3A–3B), presenting a small body, round to elongated, ventral sucker noticeably larger than oral sucker, and few large eggs in the uterus (less than 10), commonly distributed in the pretesticular or testicular region. Others were similar to specimens of *C. lynchi* reported in fishes belonging to Anostomidae from Emas Waterfalls (Cachoeira de Emas) by Kohn et al. [30] (Figs. 2A–2B; Figs. 3C–3D). These are probably all fishes belonging to the *Megaleporinus* genus, considering that the natural distribution of *Hypomasticus copelandii* Steindachner, 1875 (= *Leporinus copelandii*) ranges from the Paraíba do Sul River to Mucuri River, including Doce River and Itapemerim River, Brazil [6]). These digenean specimens exhibited an elongated body, and although the ventral sucker was larger than the oral sucker, both appeared proportionally smaller when compared to the total body length. The uterus presented numerous eggs (more than 10), exceeding the testis and/or extending to the posterior region of the body (for detailed morphological and morphometric data, see Table 3).

**Table 3:** Morphological differences between the morphometry of *Creptotrema creptotrema* and *Creptotrema lynchi*. Measurements are in micrometers.

	*C. creptotrema* (Travassos et al., 1928): original description	*C. creptotrema* (Kohn, 1984): redescription	C. creptotrema** (present study): adult in early development phase	C. creptotrema** (present study): completely developed adult	*C. lynchi* (Brooks, 1976): original description	*C. lynchi* (Kohn et al., 1985): reported in Brazilian fishes
Body length	470–540	466–622	435–824	1120–1405	850–1490	980–1590
Body width	240–280	240–390	283–379	513–663	390-670	570–680
Oral sucker	110–130*	100–132 × 126–172	83–132 × 96–172	141–206 × 175–194	156–276 × 192–336	150–180 × 180–210
Pharynx	40*	36–60 × 38–72	26–60 × 33–59	67–82 × 68–93	60-84 × 120	60–90*
Esophagus	Short	Short	Short	Long	Long	Long
Ventral sucker	160*	156–198 × 156–210	120–249 × 124–235	232–278 × 223–305	264–396 × 276–372	210–270 × 240–270
Cirrus-sac	Large; on acetabular region	132–204 × 36–42	148–156 × 29–73	258–420 × 49–79	444–660 × 80–108	240–270 × 40–60
Ovary	Round; pre-testicular; post-acetabular	48–84 × 36–66	87–114 × 82–90	151–181 × 119–182	204–264 × 216–288	130–190 × 130–210
Uterus	Pre-testicular, intercecal/Eggs between testis and genital pore	Pre-testicular, mainly intercecal/Eggs may occur in testicular region	Pre-testicular, mainly intercecal/Eggs may occur in testicular region	Mainly intercecal/Eggs may extend beyond the testis region	Pre-testicular/testicular, intercecal (may extend extracecally)	Mainly intercecal/Eggs may extend beyond the testis region
Left testis	56–96*	72–120 × 48–105	106–121 × 73–91	125–184 × 89–143	300–480 × 192–252	200–270 × 100–170
Right testis	–	72–132 × 48–92	94–121 × 89–90	153–182 × 89–111	325–540 × 180–265	210–350 × 100–150
Eggs	72–78 × 44–50	60–72 × 38–50	62–75 × 31–45	55–80 × 31–43	55–67 × 35–38	64–79 × 34–45
Number of eggs	4–5	2–5	Less than 10 eggs	More than 10 eggs	More than 10 eggs	More than 10 eggs

* Measurements of the diameter. The adult in the early development phase and completely developed adult forms of *Creptotrema creptotrema* from this study were recovered from a single host, and specimens showing both morphologies were sequenced and used in the 28S rDNA and COI mtDNA analyses; there was no intraspecific genetic divergence between these morphotypes.

Although morphological variations have been observed among the new specimens of *C. creptotrema*, there is no intraspecific genetic divergence between the sequences of these different morphotypes (see “*Molecular data and phylogenetic inference*” in the “Results” section). This may indicate that the morphological differences observed are probably due to the different stages of the ontogenetic development of the specimens. Therefore, it is suggested that the specimens of *C. creptotrema* studied by Travassos et al. [68] in the original description might represent adults in the early development phase. On the other hand, the specimens reported by Kohn et al. [30] as *C. lynchi* in anostomid fishes from Emas Waterfalls were interpreted as completely developed adult specimens belonging to *C. creptotrema*, since *C. lynchi* was originally described in toads (Table 4) from Colombia [9]. Differences in the morphology of adults in the early development phase and completely developed adults were also observed for *C. conconae* n. sp. and *C. megacetabularis* n. sp. described in this study, reinforcing this hypothesis. The differences are mainly related to the number of eggs in the uterus (fewer eggs in adults in the early development phase and eggs larger proportionally to the total body size), differences in oral sucker morphology (predominantly subspherical in adults in the early development phase) and differences in oral and ventral sucker ratio (see their respective descriptions).

**Table 4:** Valid species of *Creptotrema* Travassos, Artigas & Pereira, 1928 (Trematoda, Allocreadiidae).

Valid species	Type-host	Order, Family	Type-Locality	Reference (original description)
*Creptotrema creptotrema*	*Megaleporinus obtusidens* (Valenciennes, 1837) (= *Leporinus elongatus*)	Characiformes, Anostomidae	Emas Waterfalls (Cachoeira de Emas), Mogi-Guaçu River, São Paulo State, Brazil	[68]
*Creptotrema macrorchis* **comb. n.**	*Pachyurus bonariensis* Steindachner, 1879	Perciformes, Sciaenidae	La Plata River, Buenos Aires, Argentina	[64]
*Creptotrema platense* **comb. n.**	*Pimelodus maculatus* Lacepède, 1803 (= *Pimelodus clarias*)/*Iheringichthys labrosus* (Lütken 1874)/*Rhinodoras dorbignyi* (Kner 1855)*	Siluriformes, Pimelodidae/Siluriformes, Pimelodidae/Siluriformes, Doradidae	La Plata River, Buenos Aires, Argentina	[64]
*Creptotrema stenopteri* **comb. n.**	Charax stenopterus (Cope, 1894) (= *Asiphonichthys stenopterus*)	Characiformes, Characidae	Laguna del Sauce, Uruguay	[37]
*Creptotrema lynchi*	*Rhinella marina* (Linnaeus, 1758) (= *Bufo marinus*)	Anura, Bufonidae	San Cristobal, Atlantico, Colombia	[9]
*Creptotrema paraense*	*Pimelodus* sp.	Siluriformes, Pimelodidae	Cachimbo, Pará State, Brazil	[70]
*Creptotrema pati*	*Luciopimelodus pati* (Valenciennes, 1835)	Siluriformes, Pimelodidae	Atalaya, Partido de Magdalena, Buenos Aires Province, Argentina	[35]
*Creptotrema astyanace* **comb. n.**	*Astyanax fasciatus* (Cuvier, 1819)	Characiformes, Characidae	Loonku Creek, Región Autónoma del Atlántico del Sur, Nicaragua	[58]
*Creptotrema lamothei*	*Ageneiosus brevifilis* Valenciennes, 1840	Siluriformes, Ageneiosidae	Paraguay River, near San Antonio, Paraguay	[14]
*Creptotrema sucumbiosa*	*Tetragonopterus argenteus* Cuvier, 1816	Characiformes, Characidae	Río Aquarico, Provincia de Sucumbíos, Ecuador	[14]
				
				
				
				
*Creptotrema diagonale* **comb. n.**	*Stethaprion* cf*. erythrops* Cope, 1870	Characiformes, Characidae	Itaya River, Provincia de Mayas, Peru	[17]
*Creptotrema foliaceum* **comb. n.**	*Bryconops* cf. *caudomaculatus* (Günther, 1864)	Characiformes, Iguanodectidae	Itaya River, Provincia de Mayas, Peru	[17]
*Creptotrema totonacapanense* **comb. n.**	*Astyanax mexicanus* (de Filippi, 1853)	Characiformes, Characidae	Creek at Filipinas, Veracruz, Mexico	[53]
*Creptotrema lobata* **comb. n.**	*Brycon guatemalensis* Regan, 1908	Characiformes, Bryconidae	El Mangal Lagoon, Usumacinta River basin, Mexico	[24]
*Creptotrema tica* **comb. n.**	*Gymnotus maculosus* Albert & Miller, 1995	Gymnotiformes, Gymnotidae	Orosí River, Costa Rica	[25]
				
				
				
*Creptotrema guacurarii* **comb. n.**	*Characidium heirmostigmata* da Graça & Pavanelli, 2008	Characiformes, Crenuchidae	Arrechea Stream, Iguazu River above the waterfalls, Iguazu National Park, Misiones, Argentina	[41]
*Creptotrema conconae* **n. sp.**	*Imparfinis mirini* Haseman, 1911	Siluriformes, Heptapteridae	Upper Paraná River basin, São Paulo State, Brazil	**Present study**
*Creptotrema schubarti* **n. sp.**	*Characidium schubarti* Travassos, 1955	Characiformes, Crenuchidae	Upper Paraná River basin, São Paulo State, Brazil	**Present study**
*Creptotrema megacetabularis* **n. sp.**	*Auchenipterus osteomystax* (Miranda Ribeiro, 1918)	Siluriformes, Auchenipteridae	Upper Paraná River basin, São Paulo State, Brazil	**Present study**

* In the original description published by Szidat [64], there is no indication which of the hosts is the type-host.

Previous studies on *Creptotrema* spp. based on light microscopy may have misinterpreted the morphology of the oral sucker. SEM photomicrographs of the specimens of *C. creptotrema* collected during the present study (Figs. 1A–1C) confirm the morphology of the oral sucker. This structure presents as ventral anterior discrete horseshoe-shaped flaps and a discrete single ventrolateral muscular lobe on either side of the oral sucker, stretching from the ventral side to the lateral area, but not extending to the dorsal region, without free dorsal ends. The presence of a tegument, papillous or rugose, on the suckers described by Kohn [29] was not observed in our light microscopy and SEM analyses.

### Creptotrema conconae n. sp. (Figs. 4A–4B)

urn:lsid:zoobank.org:act:5A084499-6975-4CE4-9482-B417BED8766D

**Figure 4:**
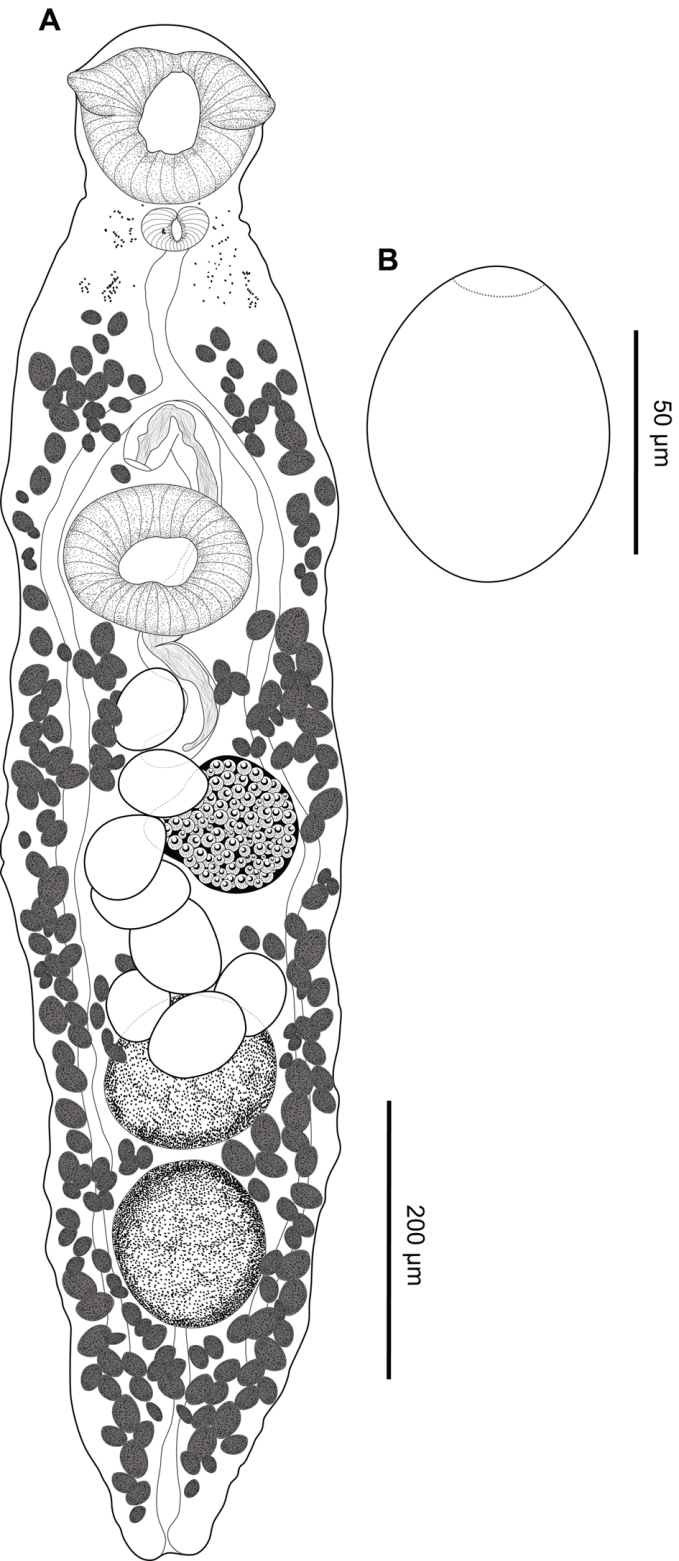
*Creptotrema conconae* n. sp. (CHIOC 39595–ventral view). A. Total view; B. Egg.

*Type-host*: *Imparfinis mirini* Haseman, 1911 (Siluriformes, Heptapteridae).

*Other hosts (present study): Cetopsorhamdia iheringi* Schubart & Gomes, 1959 (Siluriformes, Heptapteridae).

*Type-locality:* streams of the Middle Paranapanema River (23° 9′ 40.8′′ S, 48° 53′ 7.3′′ W), Avaré municipality, Upper Paraná River basin, São Paulo State, Brazil.

*Site of infection:* intestine.

*Prevalence (P), mean abundance (MA) and range*: *I. mirini* (*P* = 32.0%; *MA* = 2.4 ± 0.8 [0–28]); *C. iheringi* (*P* = 2.6%; *MA* = 0.1 ± 0.1 [0–5]).

*Material deposited*: holotype CHIOC 39595, paratypes CHIOC 39596a–g; 39597a–b, and vouchers CHIBB 645 L–651 L.

*Representative DNA sequence:* 1107 bp long sequence of the 28S rDNA gene – GenBank OK044374; 1073 bp long sequence of complete ITS – GenBank OK047367; 447 bp long sequence of COI mtDNA – GenBank OK075288, all obtained from a specimen parasitizing *I. mirini.*

*Etymology:* The epithet refers to the Dr. Maria Conceição Zocoller-Seno, retired professor at São Paulo State University, Campus of Ilha Solteira, Brazil. During her career, Dr. Zocoller-Seno worked as a researcher in the field of Animal Parasitology. Affectionately nicknamed by students and co-workers as “Concon” (in mention of the initial of her first name “Conceição”), she was a great motivator in the academic and teaching career of several students. Thus, in recognition of her performance, we name the new species as a representation of our gratitude and appreciation.

**Description**: (Based on 2 specimens stained with Mayer’s carmalumen and 8 specimens stained with Gömöri’s trichrome). Body elongated, 1463 (994–1857) long, 298 (126–421) wide, slightly constricted at pre-pharyngeal level (neck region). Remnants of eyespots present (most numerous and evident in adults in the early development phase), sparse, restricted to anterior first-third of body. Oral sucker subterminal, subspherical to funnel-shaped, with 156 (126–188) long, 161 (127–209) wide, with a single muscular lobe on either side of the oral sucker (“auricles”), with a broad base, stretching from ventrolateral to dorsolateral side and presenting free dorsal ends. Pharynx muscular, subspherical 45 (31–50) long, 59 (57–61) wide. Esophagus 141 (128–156) long. Ventral sucker pre-equatorial, 149 (116–169) long, 167 (150–179) wide, similar in size to oral sucker. Ratio of oral sucker length to ventral sucker length 1:0.9 (0.9–1.1); ratio of oral sucker width to ventral sucker width 1:1 (0.8–1.2). Intestinal ceca blind, extending to posterior end of body. Genital pore aperture slightly posterior to intestinal bifurcation, anterior to ventral sucker. Cirrus-sac well-developed, 319 (288–371) long, sinuous, passing posterior to ventral sucker, reaching the ovary posteriorly, with seminal vesicle, and unarmed and eversible cirrus. Two testes rounded, in tandem, 130 (93–179) long, 133 (100–176) wide, juxtaposed next to each other, intercecal. Ovary posterolateral to ventral sucker, pretesticular, obliquely oval to irregular in shape, with entire margin, 121 (100–175) long, 112 (87–163) wide. Vitelline follicles marginal, large, extra- and intra-cecal, not overlapping sexual structures; follicles extending from level of esophagus to posterior end of body, completely separated into two lateral fields but confluent in post-testicular region. Mehlis’ gland close to ovary. Laurer’s canal not observed. Uterus pretesticular, intra-cecal, between anterior testis and genital pore. Eggs operculate, 62 (54–69) long, 44 (33–56) wide; fewer eggs in adult specimens in the early development phase than in completely developed adults (usually > 10). Excretory pore terminal; excretory vesicle I-shaped, reaching anterior testis.

**Remarks:** *Creptotrema conconae* n. sp. was placed in this genus in the context of the current proposal that considers *Auriculostoma* synonymous to *Creptotrema*. This new species is consistent with the original generic diagnosis of *Auriculostoma* [58] and later reviewed by Razo-Mendivil et al. [53], compared to that originally described as the type-species *C. creptotrema*. The main morphological characteristic of this new species, compatible with the new diagnosis of *Creptotrema* proposed herein, is the presence of a subterminal oral sucker with a single muscular lobe on either side of the oral sucker, with a broad base, stretching from the ventrolateral to the dorsolateral side, and with free dorsal ends.

*Creptotrema conconae* n. sp. is morphologically similar to *Creptotrema astyanace* comb. n. (= *Auriculostoma astyanace*) (type-species of *Auriculostoma*), sharing characteristics such as testes in tandem, oval, and with entire margin (not lobed); uterus completely pretesticular, extending to the genital pore; cirrus-sac long and sinuous, and distribution of the vitelline follicles (both intra- and extracecal and extensively confluent in the post-testicular region) (Table 5). However, the new species and *C. astyanace* comb. n. differs by the smaller total body length and width of *C. conconae* n. sp. (on average 1463 × 298 *versus* 2900 × 488 in *C. astyanace* comb. n.); in the shape of the oral sucker, varying from subspherical to funnel-shaped in the new species *versus* funnel-shaped in *C. astyanace* comb. n.; and in the position of the ovary (post-equatorial and sinistral in the new species *versus* pre-equatorial and median in *C. astyanace* comb. n.). Although the eggs are operculated and similar in shape in both species, those observed in *C. conconae* n. sp. are larger (62 × 44) and less numerous (no more than 40 eggs in completely developed adults) *versus* smaller (55–59 long × 34–41 wide) and numerous (40–60 eggs) in *C. astyanace* comb. n. (see [58]).

**Table 5:** Morphological differences among valid species of *Creptotrema* (Trematoda, Allocreadiidae).

Valid species	Body shape	Oral lobes	Relative sucker size	Testes shape	Testes position	Vitelline follicles	Cirrus-sac posterior extent	Genital pore position	Uterus extent/Eggs
*Creptotrema creptotrema*	Round to elongated	Ventral, horseshoe-shaped, not extending to dorsal region	Oral smaller than ventral	Oval to elongated; entire margins	Symmetrical to oblique; intracecal (rarely one extracecal)	Extracecal, cecal and intracecal; separated into two lateral fields	Exceeds slightly the length of the ventral sucker	At level of ceca bifurcation	Pretesticular, inter-cecal/Eggs between testis and genital pore, but may exceed the testicular region
*Creptotrema macrorchis* **comb. n.**	Lanceolated	Stretching from the ventrolateral to the dorsolateral side	Nearly equal	Irregular; slightly lobed margins	Tandem	Extracecal in the pretesticular region; scarcely confluent in post-testicular region	Exceeds the posterior margin of the ventral sucker, reaches the ovarian level	Posterior to ceca bifurcation; preacetabular	Pretesticular, inter-cecal/Eggs between genital pore and anterior to testes
*Creptotrema platense* **comb. n.**	Elongated	Stretching from the ventrolateral to the dorsolateral side	Nearly equal	Oval; entire margins	Oblique	Extracecal in the pretesticular region; not confluent in post-testicular region	Not reaching beyond the posterior margin of the ventral sucker	Between the anterior margin of the ventral sucker and ceca bifurcation	Intertesticular, intercecal/Few eggs at the testicular level
*Creptotrema stenopteri* **comb. n.**	Elongated	Stretching from the ventrolateral to the dorsolateral side	Oral slightly smaller than ventral	Irregular; slightly lobed margins	Tandem	Extracecal in the pretesticular region; not confluent in post-testicular region	Exceeds the posterior margin of the ventral sucker, reaches the ovarian level	Anterior to the ceca bifurcation	Pretesticular, intercecal/Eggs between genital pore and anterior to testes
*Creptotrema lynchi*	Oval to elongated	Dorsolateral	Oral smaller than ventral	Elongated; entire margins	Symmetrical to slightly oblique; intracecal	Extracecal, cecal and few intercecal follicles; separated into two lateral fields	Exceeds the posterior margin of the ventral sucker	At level of ceca bifurcation	Pretesticular, intercecal or may extend extracecal/Eggs between genital pore and testes region
*Creptotrema paraense*	Elongated	Ventrolateral	Oral larger than ventral	Subspherical; entire margins	Tandem; intracecal	Irregular, Cecal and intracecal, separated into two lateral fields, partially confluent in cecal bifurcation region and confluent in post-testicular region	Exceeds the posterior margin of the ventral sucker	Anterior to ceca bifurcation	Pretesticular/Eggs between genital pore and anterior to testes
*Creptotrema pati*	Oval	Dorsolateral	Nearly equal	Elongated; entire margins	Symmetrical to oblique; extracecal	Mostly extracecal; few cecal and intracecal; separated into two lateral fields	Exceeds the posterior margin of the ventral sucker	Posterior to the ceca bifurcation; preacetabular	Inter-testicular, intercecal/Eggs between genital pore and may exceeds testes
*Creptotrema astyanace* **comb. n.**	Elongated	Stretching from the ventrolateral to the dorsolateral side	Oral slightly large than ventral	Oval; entire margins	Tandem	Extra- and intracecal; separated into two lateral fields, confluent in post-testicular region	Exceeds the posterior margin of the ventral sucker, reaches the ovarian level	Posterior to the ceca bifurcation; preacetabular	Pretesticular/Eggs between genital pore and anterior to testes
*Creptotrema lamothei*	Elongated	Ventral	Nearly equal	Irregular (lobate margin in larger individuals)	Oblique	Mostly extracecal; few cecal and intracecal; separated into two lateral fields, confluent in post-testicular region	Reaches the posterior margin of the ventral sucker	At level of ceca bifurcation	Pretesticular, intercecal/Eggs between genital pore and anterior to testes
*Creptotrema sucumbiosa*	Elongated	Ventral	Oral slightly smaller than ventral	Elongated; entire margins	Oblique	Mostly extracecal; few cecal and intracecal; separated into two lateral fields, confluent in post-testicular region	Exceeds the posterior margin of the ventral sucker, reaches the ovarian level	At level of ceca bifurcation	Intertesticular, intercecal/Eggs between genital pore and testes
*Creptotrema diagonale* **comb. n.**	Elongated	Stretching from the ventrolateral to the dorsolateral side	Oral slightly smaller than ventral	Elongated; entire margins	Oblique (contiguous or not)	Surround ceca, separated into two lateral fields, confluent in post-testicular	Exceeds the ovarian level	At level of ceca bifurcation	Intercecal, between ventral sucker and testes/Eggs intruding into testicular level
*Creptotrema foliaceum* **comb. n.**	Elongated	Stretching from the ventrolateral to the dorsolateral side	Oral larger than ventral	Irregular margins	Tandem	Extra, cecal and intracecal; separated into two-fields region, confluent in post-testicular region	Exceeds the posterior margin of the ventral sucker, reaches the ovarian level	Slightly posterior to the ceca bifurcation level	Pretesticular, intercecal/Eggs between genital pore and anterior to testes
*Creptotrema totonacapanense* **comb. n.**	Elongated	Stretching from the ventrolateral to the dorsolateral side	Oral slightly smaller than ventral	Oval; entire margins	Oblique	Extra and intracecal; separated into two lateral fields, not (or scarcely) confluent in post-testicular region	Exceeds the posterior margin of the ventral sucker, reaches the ovarian level	At level of ceca bifurcation	Intertesticular, intercecal/Eggs between genital pore and anterior testes
*Creptotrema lobata* **comb. n.**	Elongated	Stretching from the ventrolateral to the dorsolateral side	Oral slightly smaller than ventral	Deeply lobed	Tandem	Extra, cecal, and intracecal; separated into two lateral fields, confluent in post-testicular region	Exceeds the ovarian level	Slightly posterior to the ceca bifurcation level	Pretesticular, intercecal/Eggs between genital pore and anterior to testes
*Creptotrema tica* **comb. n.**	Elongated	Stretching from the ventrolateral to the dorsolateral side	Oral slightly larger than ventral	Oval; entire margins	Oblique	Extra and intracecal; separated into two lateral fields, confluent in post-testicular region	Exceeds the posterior margin of the ventral sucker, reaches the ovarian level	Anterior to the ceca bifurcation	Pretesticular, intercecal/Eggs between genital pore and posterior border of anterior testis
*Creptotrema guacurarii* **comb. n.**	Elongated	Discrete, on both sides, not extent to dorsal region	Oral slightly smaller than ventral	Elongated; entire margins	Oblique	Extra and intracecal; separated into two lateral fields, not confluent in post-testicular region	Exceeds the posterior margin of the ventral sucker, reaches the ovarian level	At level of ceca bifurcation	Pre- and inter-testicular, intercecal/Eggs between genital pore and mid-level of testes
*Creptotrema conconae* **n. sp.**	Elongated	Stretching from the ventrolateral to the dorsolateral side	Oral larger than ventral	Round; entire margins	Tandem	Extracecal; cecal and intracecal; separated into two lateral fields, confluent in post-testicular region	Exceeds the posterior margin of the ventral sucker, reaches the ovarian level	Slightly posterior to the intestinal ceca bifurcation	Pretesticular, intercecal/Eggs between genital pore and anterior to testes
*Creptotrema schubarti* **n. sp.**	Oval to elongated	Ventral (Discrete ventrolateral)	Oral smaller than ventral	Elongated; entire margins	Symmetrical to oblique	Extra, cecal and intracecal; separated into two lateral fields, not (or scarcely) confluent in post-testicular region	Exceeds the posterior margin of the ventral sucker	At level of ceca bifurcation	Intertesticular, inter-extracecal/Eggs between genital pore and may exceed the testicular region
*Creptotrema megacetabularis* **n. sp.**	Oval to elongated	Ventral, horseshoe-shaped, not extending to dorsal region	Oral smaller than ventral	Elongated; entire margins	Symmetrical	Mostly extracecal and cecal; separated into two lateral fields, not (or scarcely) confluent in post-testicular region	May exceed the posterior margin of the ventral sucker	At level of ceca bifurcation	Intertesticular, intracecal/Eggs between genital pore and rarely exceed the testicular region

*Creptotrema conconae* n. sp. is the first species of the genus described in heptapterid fishes and is morphologically similar to other species described in fishes belonging to Pimelodidae (Siluriformes) from South America, such as *Creptotrema platense* comb. n. (= *Auriculostoma platense*) and *C. paraense* from Argentina and Brazil, respectively (Table 4). The new species differs from these other congeners in its smaller body, narrower and more elongated than that described for other species of parasites of siluriform fishes. The new species presents a long and sinuous cirrus-sac, exceeding the ventral sucker posteriorly and reaching the ovary, but not longer than that described for *C. paraense* (around 400 *versus* 319 μm in the new species) *versus* a short cirrus-sac that does not reach beyond the posterior margin of the ventral sucker observed in *C. platense* comb. n.

*Creptotrema conconae* n. sp. and *C. paraense* share more morphological characteristics than *C. platense* comb. n.. The distribution of vitelline follicles is confluent in the posterior region of the testes in both species (and not confluent in *C. platense* comb. n.); the arrangement of the testes is in tandem in the new species and *C. paraense* (*versus* oblique in *C. platense* comb. n.) (Table 5). However, *C. paraense* has larger testes than the new species (210 μm *versus* 130 μm in diameter in the new species); the testes are arranged in tandem in both species, but in *C. paraense*, they do not occupy a regular position (one immediately juxtaposed to the other), as observed for the new species. In addition, the ovary of *C. paraense* has a more rounded and regular shape than that of the new species (obliquely oval to irregular in shape), and the eggs are slightly smaller (56 × 42 μm *versus* 62 × 44 μm in the new species) and more numerous than in the new species. Additionally, the size of the oral sucker is larger than that of the ventral sucker (in proportion) in *C. paraense*, whereas they are similar in size in the new species.

### *Creptotrema schubarti* n. sp. (Figs. 5A–5B)

urn:lsid:zoobank.org:act:E3F00D1D-630A-4DDA-8441-0F327FCF8012

**Figure 5:**
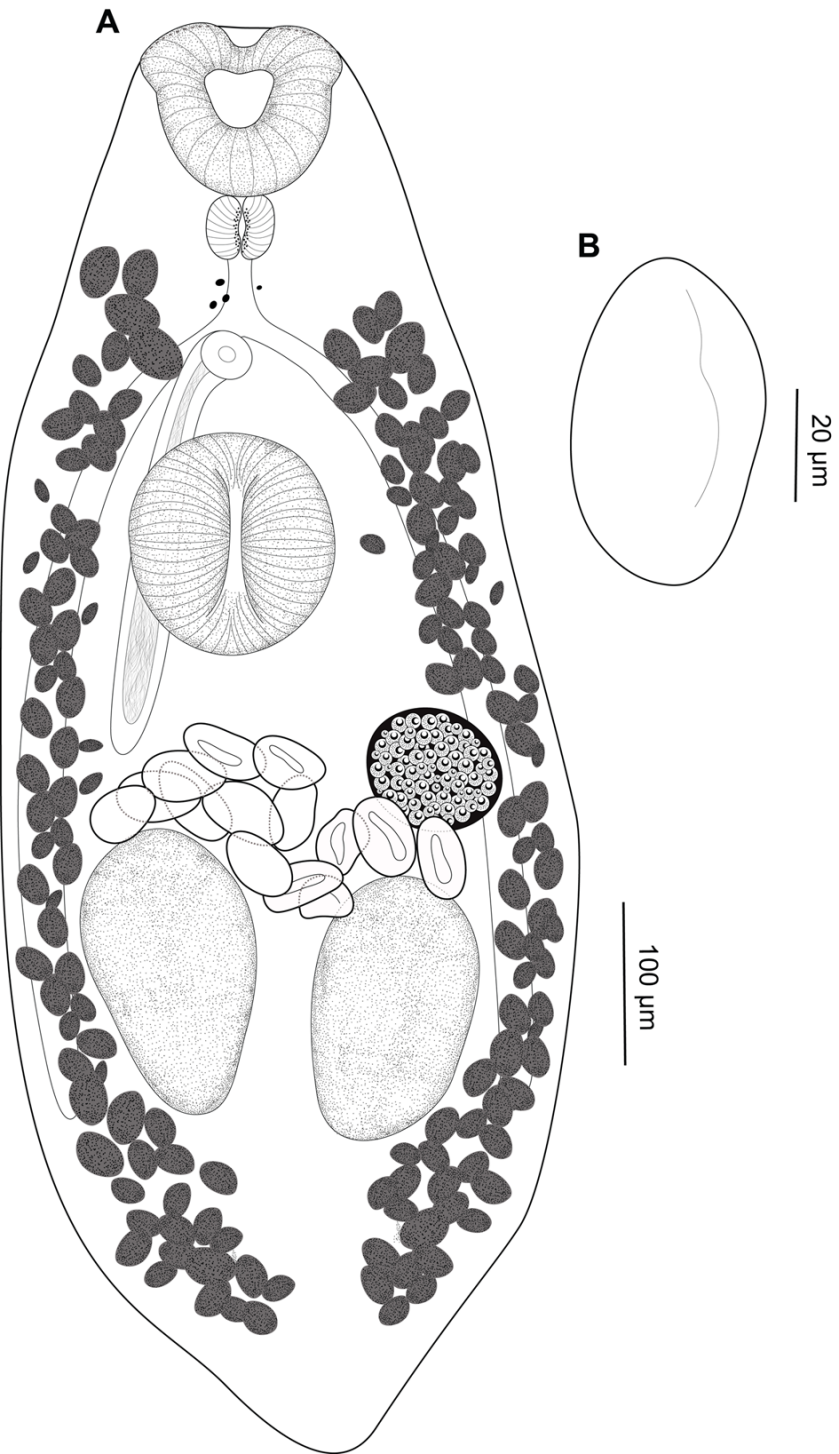
*Creptotrema schubarti* n. sp. (composite drawing) A. Total view; B. Egg.

*Type-host: Characidium schubarti* Travassos, 1955 (Characiformes: Crenuchidae).

*Type-locality:* streams of the Middle Paranapanema River, Jacutinga microbasin (23° 9′ 25.7′′ S, 48° 48′ 31.3′′ W), Avaré municipality, Upper Paraná River basin, São Paulo State, Brazil.

*Site of infection:* intestine.

*Prevalence (P), mean abundance (MA) and range*: (*P* = 5.0%; *MA* = 0.08 ± 0.05 [0–3]).

*Material deposited*: holotype CHIOC 39598 and paratypes CHIOC 39599; 39600 and 39701

*Representative DNA sequence:* 1278 bp long sequence of the 28S rDNA gene – GenBank OK044373; 442 bp long sequence of COI mtDNA – GenBank OK075293.

*Etymology:* The epithet refers to the type-host *Characidium schubarti*.

**Description**: (Based on 4 specimens stained with Mayer’s carmalumen). Body small, elongated, 802 (696–908) long, 359 (324–394) wide at midbody. Tegument smooth. Remnants of eyespots present mainly concentrated in forebody. Oral sucker subterminal, funnel-shaped, 107 (91–122) long, 133 (122–138) wide, with one pair of muscular ventrolateral lobes on both sides, forming discrete “auricles.” Ventral sucker round, pre-equatorial, 111 (71–149) long, 107 (75–138) wide. Oral sucker/ventral sucker length and width ratio 1:1 (0.8–1.2), 1:0.8 (0.6–1), respectively. Prepharynx short. Pharynx well-developed, subspherical, 60 long, 54 wide. Esophagus 75 (62–89) long. Intestinal ceca blind, extending to posterior end of body. Genital pore median, at level of intestinal bifurcation in forebody. Cirrus unarmed, eversible. Cirrus-sac well-developed, 335 (250–420) long, exceeding length of ventral sucker; seminal vesicle and pars prostatica not observed. Two testes in posterior half of body, 142 (104–179) long, 108 (82–122) wide, oval to elongated, with entire margins, symmetrical, or slightly oblique, cecal, or intercecal. Ovary oval, pretesticular, posterolateral to ventral sucker, 140 long, 122 wide, with entire margins. Mehlis’ gland and Laurer’s canal not observed. Vitelline follicles large, dense, extending from level of oral sucker to posterior end of body, extracecal, cecal, and few intra-cecal, not (or scarcely) confluent in post-testicular region. Uterus extending between genital pore and may exceed the testis region; loops may extend into extracecal area. Eggs large, 55 (49–61) long, 37 (28–50) wide. Excretory pore terminal; excretory vesicle I-shaped, reaching testis region.

**Remarks:** *Creptotrema schubarti* n. sp. is morphologically more similar to the recently described species *C. guacurarii* comb. n.. Both species were described in fishes belonging to *Characidium* from Brazil and Argentina, respectively. These species share features such as the presence of remnants of eyespots in the forebody (more numerous and evident in young specimens), oral sucker subterminal, with one pair of discrete muscular oral lobes on both sides. However, the new species is smaller (802 [696–908] μm long *versus* 1254 [1084–1439] μm long in *C. guacurarii* comb. n.), and the body shape is usually oval (*versus* well elongated in *C. guacurarii* comb. n.). The testes of *Creptotrema schubarti* n. sp. are symmetrical or slightly oblique, intercecal, but may overlap the cecal region, while in *C. guacurarii* comb. n., they are clearly oblique and strictly occupy the intercecal region. In *Creptotrema schubarti* n. sp., the uterus extends between genital pore and may exceed the testes region, and the loops may extend into the extracecal area; in *C. guacurarii* comb. n., the uterus extends between the genital pore and mid-level of testes, with loops extending into pre- and inter-testicular regions, intercecal. The post-testicular space length is longer in *C. guacurarii* comb. n., representing 27 (25–30) percent of body length, while in the new species this space represents approximately 15% of the body length. Both species presented few eggs; however, they were smaller in the new species (55 [49–61] μm long, 37 [28–50] μm wide *versus* 67 [65–70] μm long, 40 [38–42] μm wide in *C. guacurarii* comb. n.).

In addition, *Creptotrema schubarti* n. sp. can be distinguished from other congeners that have symmetrical to oblique testes (*C. creptotrema*, *C. pati*, *C. lynchi*, and *C. megacetabularis* n. sp.) by the distribution of eggs in the uterus. The eggs may occupy the region between the genital pore and may exceed the testes region, extending into the extracecal area in the new species, whereas, in other congeners, the eggs are distributed preferentially in the pretesticular and inter-testicular regions (as in *C. lynchi*), but may extend posteriorly to the testes, but typically not extracecal (as in *C. creptotrema* and *C. megacetabularis* n. sp.). In *C. pati*, the eggs are also distributed in the uterus in the inter-testicular region and may extend to the post-testicular region, but the uterus is intercecal (not extending to the extracecal region as in the new species), and the testes are extracecal (*versus* intra-cecal in the new species) (Table 5).

### *Creptotrema megacetabularis* n. sp. (Figs. 6A–6B)

urn:lsid:zoobank.org:act:6EE849E3-74C9-4814-9DDE-94A2DED3A1DB

**Figure 6:**
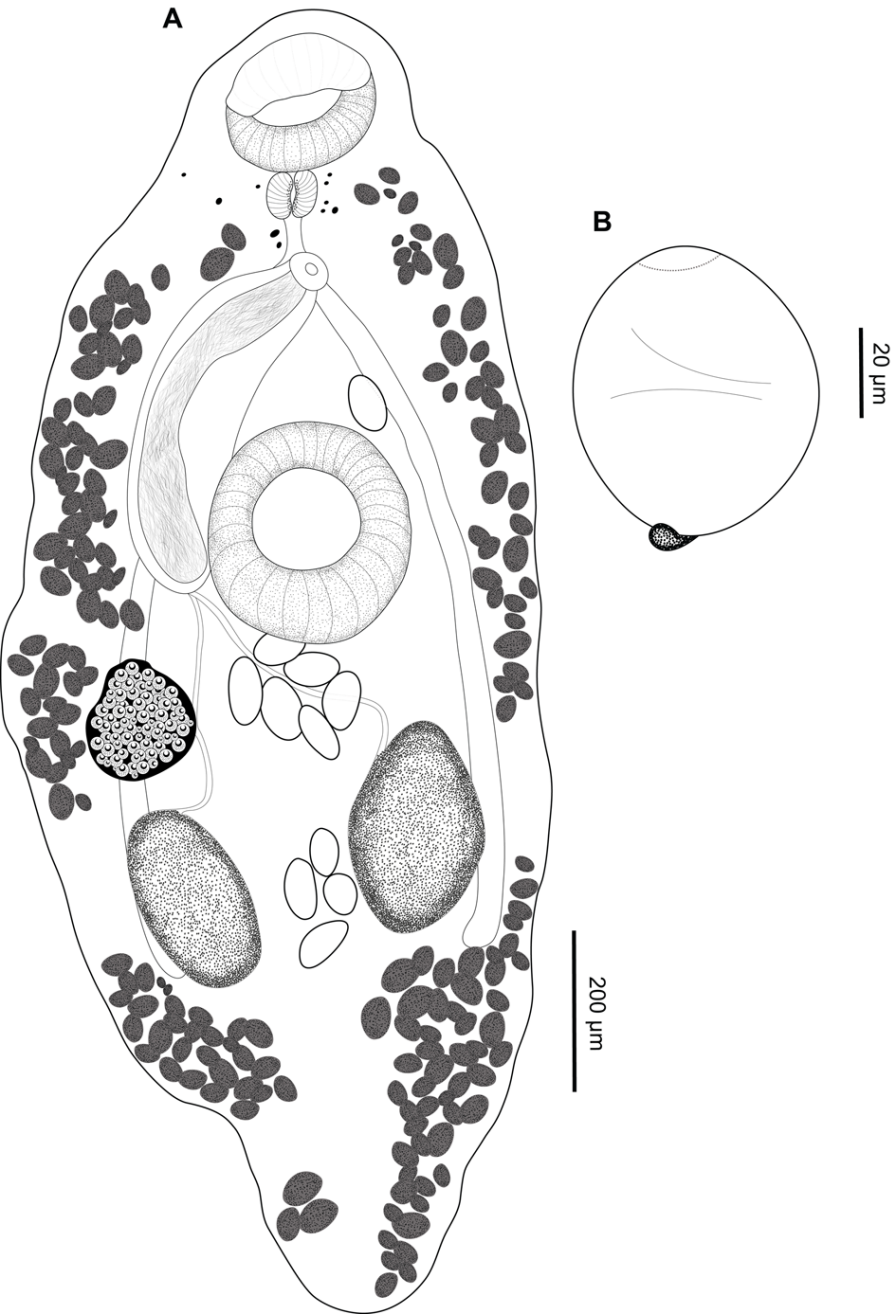
*Creptotrema megacetabularis* n. sp. (composite drawing). A. Total view; B. Egg.

*Type-host: Auchenipterus osteomystax* (Miranda Ribeiro, 1918) (Siluriformes: Auchenipteridae).

*Type-locality*: Aguapeí River, a tributary of the Paraná River (21° 03′ 03.16′′ S, 51° 45′ 58.16′′ W), Castilho municipality, Upper Paraná River basin, São Paulo State, Brazil

*Site of infection:* intestine.

*Prevalence (PA), mean abundance (MA) and range*: (*P* = 31.25%; *MA* = 1.0 ± 0.45 [0–13]).

*Material deposited*: holotype CHIOC 39703; paratypes CHIOC 39704 a–b; 39705 and 39706 a–h.

*Representative DNA sequence:* 1216, 1065, 1129, and 1078 bp long sequences of the partial 28S rDNA gene – GenBank OK044375, OK044376, OK044377 and OK044378, respectively; 1193 bp long sequence of complete ITS – GenBank OK047366; 437 bp long sequence of COI mtDNA – GenBank OK075289.

*Etymology:* The epithet refers to the presence of a remarkable ventral sucker larger than the oral sucker, with oral sucker/ventral sucker length and width ratio 1:1.9 (1.5–2.2) and 1:1.7 (1.4–1.9), respectively.

**Description:** (Based on 2 specimens stained with Mayer’s carmalumen and 11 specimens stained with Gömöri’s trichrome). Body round to elongated, 1188 (930–1552) long, 525 (332–625) wide. Tegument smooth. Few remnants of eyespots present in forebody (more evident in young forms). Oral sucker subterminal, rounded, 127 (101–151) long, 144 (115–190) wide, with a muscular ventral anterior discrete horseshoe-shaped structure, with a reduced single muscular lobe on either side of oral sucker (difficult to observe in some mounted specimens). Ventral sucker round, equatorial, perceptibly larger than oral sucker, 222 (190–270) long, 213 (160–247) wide. Ratio of oral sucker length to ventral sucker length 1:1.9 (1.5–2.2); ratio of oral sucker width to ventral sucker width 1:1.7 (1.4–1.9). Prepharynx short. Pharynx subspherical 45 (37–56) long, 58 (45–68) wide. Esophagus 53 (52–54) long. Intestinal ceca blind, extending to posterior end of body, not exceeding posterior margin of testes. Genital pore median, at level of intestinal bifurcation in forebody. Cirrus unarmed, eversible. Cirrus-sac well-developed, 428 (393–484) long, usually exceeding length of ventral sucker; seminal vesicle and pars prostatica not observed. Testes in posterior half of body, 169 (105–213) long, 112 (78–134) wide, oval, entire margins, symmetrical, intercecal. Ovary 145 (116–185) long, 110 (92–134) wide, posterolateral to ventral sucker, post-equatorial, pretesticular, entire margins. Vitelline follicles small, extending from level of pharynx to posterior end of body, mainly extracecal and cecal, completely separated into two lateral fields, not confluent in post-testicular region. Mehlis’ gland close to ovary. Laurer’s canal not observed. Uterus intercecal, inter-testicular, between testis region (rarely exceeds the testicular zone) and genital pore. Eggs large, operculate, 68 (57–78) long, 47 (34–58) wide, with a knob opposite to the operculum, usually concentrated at testicular level, mainly in intra-cecal region. Excretory pore terminal in hind body region; excretory vesicle I-shaped, reaching testis zone.

**Remarks:** This species was erroneously identified as *C. creptotrema* by Yamada et al. [73]; however, it is herein recognized and described as a new species. As previously reported for *C. creptotrema*, *C. megacetabularis* n. sp. also shows variation in the morphology of specimens according to their ontogenetic development (adults in the early development phase are relatively smaller, have fewer eggs in the uterus, and the difference in the oral/ventral sucker is proportionally more evident than in completely developed adults). However, there was no intraspecific genetic divergence within the sequences of the morphotypes identified as adults in the early development phase and completely developed adults (see “*Molecular data and phylogenetic inference*” section).

The morphology of this new species resembles that of other congeners that also have symmetrical to oblique testes: *C. creptotrema*, *C*. *pati*, *C. lynchi*, *C. guacurarii* comb. n., and *C. schubarti* n. sp. (Table 5). However, the new species can be distinguished by the following features: the presence of a large ventral sucker, with almost twice the size of the oral sucker (oral/ventral sucker ratio nearly 1:2) (this difference may be even more evident in adults in the early development phase), well evidenced by the proportion of the size and width of the body of the parasite, and eggs with a knob opposite to the operculum.

Although there are some similarities in the morphology of this new species and *C. lynchi* reported from toads from Colombia [9], differences in the average measurements and proportions of these structures can be observed. The testes are elongated in both species, but they are smaller (169 × 112 μm on average) and symmetrical in *C. megacetabularis* n. sp. *versus* larger (300–480 × 192–252 μm) and symmetrical to oblique in *C. lynchi*; the cirrus-sac of the new species is smaller, approximately 428 μm *versus* 444–660 μm in *C. lynchi.*

The testes are intra-cecal in this new species, *C. creptotrema*, *C. lynchi*, *C. guacurarii* comb. n., and *C. schubarti* n. sp. (sometimes cecal), but they are extracecal in *C. pati*. The total length of the body (on average) of the new species (1188 μm) was similar to that observed in *C. guacurarii* comb. n. (1254 μm) and in the completely developed *C. creptotrema* adults analyzed in the present study (1248 μm), but was larger than that in *C. creptotrema* specimens analyzed in the original description (562 μm) and *C. schubarti* n. sp. (802 μm).

### Molecular data and phylogenetic inference

We successfully obtained eight partial sequences of the 28S rDNA: two of *C. creptotrema* (GenBank accession numbers OK044371 [adult in the early development phase] and OK044372 [completely developed adult], one of *C. conconae* n. sp. (GenBank accession number OK044374), one of *C. schubarti* n. sp. (GenBank accession number OK044373), and four of *C. megacetabularis* n. sp. (GenBank accession numbers OK044375–OK044376 [adult in the early development phase], and OK044377–OK044378 [completely developed adult]). Two sequences of the complete ITS were obtained: one of *C. conconae* n. sp. (GenBank accession number OK047367) and one of *C. megacetabularis* n. sp. (GenBank accession number OK047366). For the COI mtDNA, we obtained six complete sequences: three of *C. creptotrema* (GenBank accession numbers OK075290–OK075292 [adult in the early development phase] and OK075291 [completely developed adult]), one of *C. conconae* n. sp. (GenBank accession number OK075288), one of *C. schubarti* n. sp. (GenBank accession number OK075293), and one of *C. megacetabularis* n. sp. (GenBank accession number OK075289).

The 28S rDNA final alignment was 1038 bp long. The I_ss_ indicated no saturation in either the transitions or transversions; the critical index of substitution saturation (I_ss.c_) values were above the I_ss_ values.

**Figure 7:**
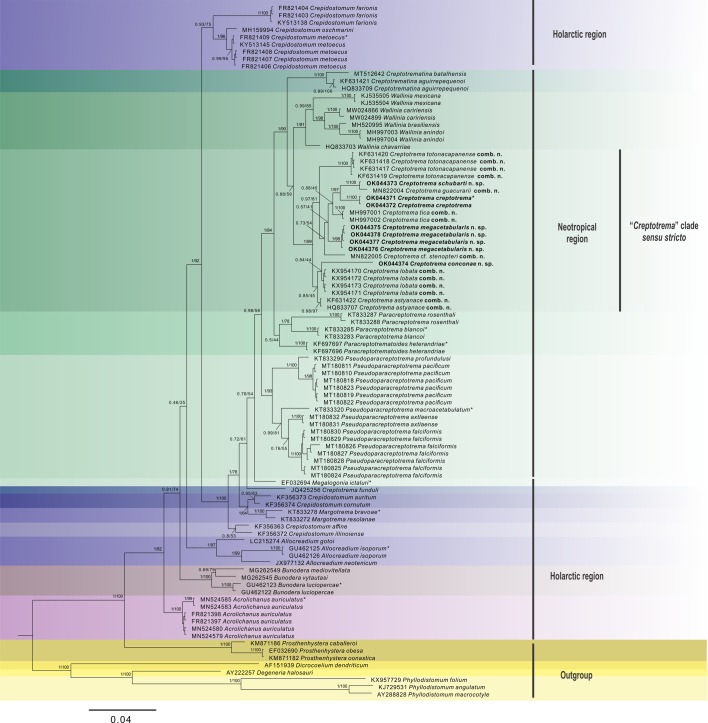
Bayesian topology based on 28S rDNA sequences of digenean parasites. GenBank accession numbers precede species names. New sequences obtained for the present study are in bold. *Prosthenhystera* spp. (Callodistomidae), *Dicrocoelium dendriticum* (Dicrocoeliidae), *Degeneria halosauri,* and *Phyllodistomum* spp. (both Gorgoderidae) were used as an outgroup. Support values are above nodes: posterior probabilities and bootstrap scores greater than 0.90 and 70, respectively, were considered well supported. The asterisk indicates the type-species of each genus. The branch length scale bar indicates the number of substitutions per site (information about the sequences are shown in the Table 2).

The 28S rDNA ML and BI phylogenetic analyses presented identical topologies, with most clades being well supported. Both analyses also recovered the Allocreadiidae as monophyletic. In the final 28S rDNA phylogenetic reconstruction (Fig. 7), two main clades were observed within the Allocreadiidae clade (pp = 1; bootstrap = 82). One comprised *Acrolichanus auriculatus* (Wedl, 1858) Ward, 1917, parasites of sturgeons (Acipenseriformes) from the Holarctic region (pp = 1; bootstrap = 100), and the other included all the other sequences of allocreadiids from Neotropical and Holarctic regions (pp = 0.91; bootstrap = 74). The clade composed of allocreadiids from the Neotropical region comprised sequences of the genera *Creptotrematina*, *Wallinia*, *Creptotrema* (*sensu stricto* clade, including “*Auriculostoma*” spp.), *Paracreptotrema*, *Paracreptotrematoides*, and *Pseudoparacreptotrema*; the North American *Megalogonia ictaluri* Surber, 1928 appears as a sister taxon of this clade. *Creptotrema funduli* from Mississippi (USA) is basal to the clade composed of allocreadiids from the Neotropical region and *M. ictaluri*.

The newly generated sequences of *Creptotrema* spp. formed a clade referred to as *Creptotrema sensu stricto* (pp = 1; bootstrap = 99), with *Wallinia* spp. recovered as its sister group. The *Creptotrema sensu stricto* clade included the sequences of the type-species *C. creptotrema*, the sequences of the new species described herein, and all sequences previously deposited in the GenBank as *Auriculostoma* spp.. *Creptotrema funduli*, the only sequence of the genus previously deposited in GenBank, was not positioned within the *Creptotrema sensu stricto* clade, suggesting that this species might not belong to *Creptotrema* and should be reevaluated (Fig. 7).

Among the *Creptotrema sensu stricto* clade, *C. schubarti* n. sp. and *C. guacurarii* comb. n. clustered together, both recovered from hosts belonging to *Characidium* from Brazil and Argentina, respectively. These species were closely related to *C. creptotrema*, forming a sister group of *Creptotrema tica* comb. n. (= *A. tica*) (all species preferentially parasitize characiform fishes). *Creptotrema megacetabularis* n. sp. (from siluriforms belonging to Auchenipteridae) appeared as a sister taxon in this group. *Creptotrema conconae* n. sp. (the only species that parasitizes heptapterids) appeared as a sister clade of this group, clustering together with *C. astyanace* comb. n. and *C. lobata* comb. n.

*Crepidostomum* spp. were distributed in three different clades, one of them well-supported (pp = 0.93; bootstrap = 75) and comprised of sequences of *Crepidostomum metoecus* (Braun, 1900) (type-species), *Crepidostomum farionis* (Müller, 1780) Lühe, 1909 and *Crepidostomum oschmarini* Zhokhov & Pugacheva, 1998, parasites of salmoniform (*C. metoecus* and *C. farionis*) and cypriniform (*C. oschmarini*) fishes, and referred to herein as the clade *Crepidostomum sensu stricto*. Another clade (pp = 0.95; bootstrap = 63) was positioned as a sister group of *Margotrema* spp. from Mexico and was composed of *Crepidostomum auritum* (MacCallum, 1919) and *Crepidostomum cornutum* (Osborn, 1903) Stafford, 1904, both parasites of perciform fishes from Mississippi, USA. The last clade (pp = 0.8; bootstrap = 53) included *Crepidostomum affine* Tkach, Curran, Bell & Overstreet, 2013 and *Crepidostomum illinoiense* Faust, 1918*,* both parasites of hiodontiform fishes from the USA (Fig. 7).

For the partial 28S rDNA, the interspecific genetic divergences found among the sequences of the new species and *C. creptotrema* (type-species) varied from 2.1 to 5.2% (21–49 bp); among *Creptotrema* spp. (except *C. funduli*) and species previously described as *Auriculostoma*, it varied from 0.4% to 5.0% (6–49 bp); from *Creptotrema* spp. (including the new sequences and *Auriculostoma* spp. reassigned herein) and *Wallinia* spp. (its sister clade), it varied between 2.9% and 6.9% (39–63 bp). There was no intraspecific genetic divergence within the sequences of *C. creptotrema* and *C. megacetabularis* n. sp. that presented differences between their morphology. For the other species of this genus, only one sequence per species was available; therefore, intraspecific comparisons could not be inferred. See Supplementary Table S1 for information on the genetic divergence values using the 28S rDNA gene.

The final COI mtDNA alignment was 352 bp long. The I_ss_ indicated no saturation in either the transitions or transversions, and the critical index of substitution saturation (I_ss.c_) values were above the I_ss_ values.

**Figure 8:**
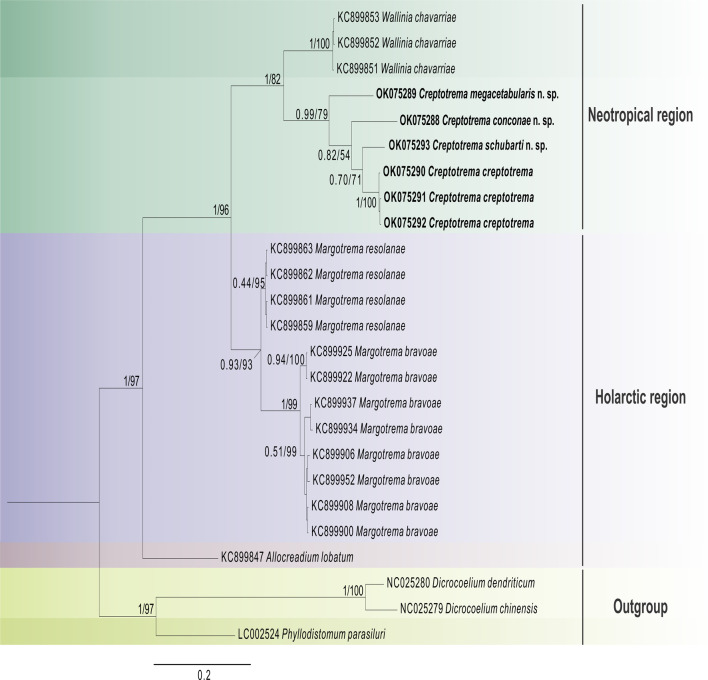
Bayesian topology based on COI mtDNA of digenean parasites. GenBank accession numbers precede species names. New sequences obtained for the present study are highlighted in bold. *Phyllodistomum prasiluri* (Gorgoderidae) and *Dicrocoelium* spp. (Dicrocoeliidae) were used as an outgroup. Support values are above nodes: posterior probabilities and bootstrap scores greater than 0.90 and 70, respectively, were considered well supported. The branch length scale bar indicates the number of substitutions per site (information about the sequences is shown in Table 2).

Both ML and BI phylogenetic analyses of COI mtDNA alignment converged with similar topologies. In the final COI mtDNA phylogenetic reconstruction (Fig. 8), all *Creptotrema* spp. sequences grouped together in a well-supported monophyletic clade (pp = 0.99; bootstrap = 79) and were positioned as a sister group of *Wallinia chavarriae* Choudhury, Daverdin & Brooks, 2002 (pp = 1; bootstrap = 82). *Creptotrema schubarti* n. sp. appeared to be the most closely related species to *C. creptotrema* (pp = 0.70; bootstrap = 71), although poorly supported in the BI analyses (Fig. 8).

For the mtCOI gene, the genetic divergences between all *Creptotrema* spp. varied from 6.6 to 16.4% (21–45 bp) (see Supplementary Table S2). No intraspecific genetic divergence was observed between the three *C. creptotrema* sequences; for the other species of this genus, only one specimen was sequenced.

## Discussion

Information regarding the species diversity of allocreadiids from Neotropical freshwater fishes has increased recently as a result of integrative taxonomic approaches involving morphological and phylogenetic analyses [25]. However, there is still a shortage of information about these digeneans that may generate inconsistencies in species delimitations (*e.g.*, *Crepidostomum*, *Paracreptotrema*, *Megalogonia*, and herein demonstrated for species of *Auriculostoma* and *Creptotrema*), their relationships, and host-parasite associations [17, 24, 25, 49].

The accurate morphological analyses of type-specimens of *Creptotrema* deposited in museums and the phylogenetic positioning among isolates provided in this study allowed us to formally describe three new species and to amend the diagnosis of *Creptotrema.* We observed intraspecific morphological variation among specimens of *C. creptotrema* collected in this study, *C. conconae* n. sp., and *C. megacetabularis* n. sp. However, the absence of intraspecific genetic divergence between the sequences of the different morphotypes of each species (reported as *C. creptotrema* and *C. megacetabularis* n. sp., herein referred to as adults in the early development phase and completely developed adults), reinforces the hypothesis that these morphological differences represent distinct stages of ontogenetic development of these species.

Although we have found low interspecific genetic divergences between the partial 28S rDNA sequences of *Creptotrema schubarti* n. sp. and *C. guacurarii* comb. n. (0.4%, 6 bp), the set of morphological differences presented herein supports the erection of the new species. These are the only sequences of the *Creptotrema* clade *sensu stricto* generated from parasites of fishes belonging to the genus *Characidium* from Brazil and Argentina, respectively, which may have contributed to the low divergence observed. The 28S rDNA represents a highly conserved fragment and is not an ideal molecular marker for the delimitation of species or for making inferences about interspecific divergences. Unfortunately, to date, there are no other deposited sequences of *C. guacurarii* comb. n. from other molecular markers useful for species delimitation, such as the mtCOI gene, which would enable a more precise analysis of molecular divergences between them. However, a comprehensive and meticulous analysis of the morphological features of both species provided important evidence for their delimitation.

The molecular evidence demonstrated herein identified *Auriculostoma* as a synonym of *Creptotrema*, and supports the monophyly of the clade *Creptotrema sensu stricto*. Based on our morphological analyses, it is possible to affirm that the morphology of the oral sucker and muscular lobes (“auricles”) is highly variable among *Creptotrema* spp. (considering the species previously allocated in *Auriculostoma*), and represents a shared feature among other “papillose” allocreadiids as a result of convergent evolution. Therefore, this character should not be considered sufficient to delimitate and distinguish species of *Creptotrema* or genera of “papillose” allocreadiids, since this character arose or was secondarily lost several times during the evolutionary history of the group [46]. We recommend the use of different tools for consistent morphological and molecular analyses to diagnose the species.

According to our 28S rDNA phylogenetic inference (see *Results* section), the sequence of *C. funduli* was not grouped within the *Creptotrema sensu stricto* clade. Considering that this sequence was not sourced from the type-host or type-locality [15, 46], we hypothesize that the specimen might not belong to *Creptotrema* and may represent a new genus yet to be properly described, corroborating the paraphyly of the genus recently proposed by Pérez-Ponce de León et al. [46]. After analyzing the types deposited in museums and based on our molecular data, *C. funduli* is proposed herein as *species inquirenda*; future studies must be conducted to resolve its taxonomic status.

Although the morphology of *C. funduli* resembles that of other species assigned to *Creptotrema*, this species shows conspicuous differences in its diagnostic characteristics. *Creptotrema funduli* was considered incorrectly assigned to this genus by Manter [38]; however, the species continued to be allocated erroneously to this genus [26]. Manter [38] also analyzed 10 co-types and observed conspicuous finger-like lobe projects inward from the dorsal wall of the oral cavity, which is not compatible with the original description of *Creptotrema*, corroborating the molecular analysis here.

*Creptotrema mulleri* Coil & Kuntz, 1960, the only species supposedly belonging to this genus and described outside the American continent, was considered a synonym of *Crepidostomum farionis* [38]. Manter [38] examined the type and paratype specimens of *C. mulleri* and observed four partially retracted papillae (= lobes) on the dorsal surface of the oral sucker. Considering this morphological feature and the fact that *C. mulleri* was described from specimens collected in salmonids (trout) from Turkey, its supposed relationship with *Creptotrema* spp. was not accepted in this study.

*Creptotrema* is now one of the most widely distributed genera of trematodes across the Americas, represented by 19 valid species of parasites of freshwater teleosts and anurans (15 from South America, two from Central America, and two from North America), and South America seems to be an important center of diversification for these allocreadiids. Excluding the nominal species *C. funduli*, which requires a taxonomic and systematic review, it is possible that the Neotropical species of *Creptotrema* (*Creptotrema sensu stricto* clade) comprises a separate monophyletic group as suggested by Choudhury et al. [13]. Although the definitive hosts of *Creptotrema* spp. are preferentially freshwater fishes belonging to multiple orders (Characiformes, Gymnotiformes, Perciformes, and Siluriformes), excluding *C. lynchi* as originally described in toads, there seems to be a close relationship between this lineage of digeneans and characiforms, in which 10 of the 19 valid species have been reported (Table 4).

Among allocreadiids from the Holarctic region, our results using the 28S rDNA gene corroborate the validity of the genus *Megalogonia* as proposed by Curran et al. [16], and the paraphyletic condition of the *Crepidostomum* observed by Atopkin and Shedko [3], Pérez-Ponce de León et al. [45], Petkevičiūtė et al. [49], and Atopkin et al. [5].

*Crepidostomum* was originally described to include parasites of salmoniforms, but some species have also been reported in cyprinodontiforms, hiodontiforms, perciforms, gadiforms, and scorpaeniforms ([21] and references cited in Table 2). The validity of many species of *Crepidostomum* has been questioned because few characters can be used for species delimitation, some of which are shared among other “papillose” allocreadiids, and some of the available molecular data are not supported by voucher specimens [21, 49, 66]. In our analyses using the 28S rDNA gene, the clade composed of *Margotrema* spp., *C. cornutum,* and *C. auritum* may indicate a closer relationship among these parasites (all sequences from the USA and Mexico), indicating the need for supplementary studies regarding their morphology and new molecular data to investigate whether they may belong to the same genus or if they may be erected in a new genus.

Considering that South America is exceedingly rich in freshwater fish fauna (approximately 5160 species, according to Reis et al. [57]), less than 5% of these potential hosts have been examined for parasites, making it difficult to predict the total trematode diversity [12]. This percentage is much higher in North America (45%) and Central America (32%); however, these subcontinents comprise a lower species richness of freshwater fishes (1213 and 299 species, respectively) than South America. This highlights the need for further parasitological surveys throughout the Americas to improve knowledge about the parasite species richness and host-parasite relationships, especially for South America, along with the use of different tools for morphological analyses (*e.g.*, light microscopy and SEM) and molecular studies (employment of different markers for phylogenetic analyses). Our results describe new species, molecular data, and important information, increasing our understanding of the relationships among allocreadiids, especially *Creptotrema*, contributing substantially to clarifying the phylogeny of this genus.

## Conflict of interest

The authors declare that they do not have any conflict of interest.

## Supplementary Material

The supplementary material of this article is available at https://www.parasite-journal.org/10.1051/parasite/2021065/olm *Table S1*. Nucleotide divergence (*p*-distance expressed in %) estimated for the 28S rDNA among *Creptotrema* spp. and selected digeneans *Table S2*. Nucleotide divergence (*p*-distance expressed in %) estimated for the mitochondrial cytochrome c oxidase I (COI mtDNA) among *Creptotrema* spp. and selected digeneans

## Financial support

The authors would like to thank FAPESP (The São Paulo Research Foundation), CAPES (Coordination for the Improvement of Higher Education Personnel), CNPq (National Council for Scientific and Technological Development), and Pro-Rectory of Research (PROPe – UNESP) for financial and scientific support, and the post-graduate scholarships granted to L.F. (CAPES/PNPD 17/2016); A.A. (FAPESP 2018/25554-2); A.C.Z. (FAPESP 2016/07829-9); P.O.F.Y. (FAPESP 2013/25786-7); and M.B.E (CNPq 140873/2017-1); R.J.S. is supported by FAPESP (2016/50377-1) and CNPq (309125/2017-0; PROTAX: 440496/2015-2).
