# Supplementary material for: Three new species of Creptotrema (Trematoda, Allocreadiidae) with an amended diagnosis of the genus and reassignment of Auriculostoma (Allocreadiidae), based on morphological and molecular evidence
Source: Parasite. 2021 Oct 13;28:69. doi: 10.1051/parasite/2021065 (PMC8513519; doi:10.1051/parasite/2021065)
Supplement: Supplementary file 2 — Table S2. Nucleotide divergence (p-distance expressed in %) estimated for the mitochondrial cytochrome c oxidase I (COI mtDNA) among Creptotrema spp. and selected digeneans [file parasite-28-69-s2.pdf]

Supplementary material - Table S2. Nucleotide divergence (p-distance expressed in %) estimated for the mitochondrial cytochrome c oxidase I (COI mtDNA) among *Creptotrema* spp. and selected digeneans

	1	2	3	4	5	6	7	8	9	10	11	12	13	14	15	16	17	18	19	**20**	**21**	**22**	**23**	**24**	**25**
1 NC025280 *Dicrocoelium dendriticum*																									
2 NC025279 *Dicrocoelium chinensis*	8.8																								
3 LC002524 *Phyllodistomum parasiluri*	31.0	34.1																							
4 KC899847 *Allocreadium lobatum*	34.9	38.0	33.2																						
5 KC899863 *Margotrema resolanae*	33.8	35.1	28.7	26.0																					
6 KC899862 *Margotrema resolanae*	33.8	35.1	28.7	26.0	0.0																				
7 KC899861 *Margotrema resolanae*	33.8	35.1	28.7	26.0	0.0	0.0																			
8 KC899859 *Margotrema resolanae*	33.8	35.1	28.7	26.0	0.0	0.0	0.0																		
9 KC899925 *Margotrema bravoae*	37.7	36.4	33.5	34.4	8.2	8.2	8.2	8.2																	
10 KC899922 *Margotrema bravoae*	37.7	36.4	33.5	34.4	8.2	8.2	8.2	8.2	0.0																
11 KC899937 *Margotrema bravoae*	37.6	37.7	34.7	34.6	8.6	8.6	8.6	8.6	2.1	2.1															
12 KC899934 *Margotrema bravoae*	37.6	37.7	35.4	34.6	9.0	9.0	9.0	9.0	2.4	2.4	0.3														
13 KC899906 *Margotrema bravoae*	38.3	37.7	34.7	33.8	8.2	8.2	8.2	8.2	1.8	1.8	1.5	1.8													
14 KC899952 *Margotrema bravoae*	38.3	37.0	33.4	33.0	8.2	8.2	8.2	8.2	1.8	1.8	1.5	1.8	0.6												
15 KC899908 *Margotrema bravoae*	39.0	37.7	34.1	33.8	7.8	7.8	7.8	7.8	1.5	1.5	1.2	1.5	0.3	0.3											
16 KC899900 *Margotrema bravoae*	39.0	37.7	34.1	33.8	7.8	7.8	7.8	7.8	1.5	1.5	1.2	1.5	0.3	0.3	0.0										
17 KC899853 *Wallinia chavarriae*	34.5	39.1	37.4	36.3	16.4	16.4	16.4	16.4	19.1	19.1	18.2	17.7	19.6	18.7	19.1	19.1									
18 KC899852 *Wallinia chavarriae*	34.5	39.1	37.4	36.3	16.4	16.4	16.4	16.4	19.1	19.1	18.2	17.7	19.6	18.7	19.1	19.1	0.0								
19 KC899851 *Wallinia chavarriae*	34.5	39.1	37.4	36.3	16.4	16.4	16.4	16.4	19.1	19.1	18.2	17.7	19.6	18.7	19.1	19.1	0.0	0.0							
**20 *Creptotrema megacetabularis* n. sp.**	30.6	39.4	34.5	38.6	20.3	20.3	20.3	20.3	25.7	25.7	26.3	26.9	25.1	25.1	25.7	25.7	15.7	15.7	15.7						
**21 *Creptotrema conconae* n. sp.**	35.2	36.6	31.7	35.7	23.5	23.5	23.5	23.5	25.6	25.6	26.3	26.9	25.7	25.7	26.3	26.3	21.1	21.1	21.1	**16.4**					
**22 *Creptotrema schubarti* n. sp.**	30.5	34.1	30.6	33.9	16.9	16.9	16.9	16.9	21.2	21.2	22.2	22.7	22.2	22.2	22.7	22.7	16.5	16.5	16.5	**12.4**	**12.5**				
**23 *Creptotrema creptotrema***	31.7	35.5	29.6	31.1	16.5	16.5	16.5	16.5	22.5	22.5	24.1	23.5	23.0	23.0	23.5	23.5	16.9	16.9	16.9	**13.3**	**13.2**	**6.8**			
**24 *Creptotrema creptotrema***	32.0	35.7	31.7	33.0	18.1	18.1	18.1	18.1	23.6	23.6	25.2	24.6	24.1	24.1	24.6	24.6	18.0	18.0	18.0	**13.3**	**12.7**	**6.6**	**0.0**		
**25 *Creptotrema creptotrema***	32.0	35.7	31.7	33.0	18.1	18.1	18.1	18.1	23.6	23.6	25.2	24.6	24.1	24.1	24.6	24.6	18.0	18.0	18.0	**13.3**	**12.7**	**6.6**	**0.0**	**0.0**	
