# Supplementary material for: Three new species of Creptotrema (Trematoda, Allocreadiidae) with an amended diagnosis of the genus and reassignment of Auriculostoma (Allocreadiidae), based on morphological and molecular evidence
Source: Parasite. 2021 Oct 13;28:69. doi: 10.1051/parasite/2021065 (PMC8513519; doi:10.1051/parasite/2021065)
Supplement: Supplementary file 1 — Table S1. Nucleotide divergence (p-distance expressed in %) estimated for the 28S rDNA among Creptotrema spp. and selected digeneans [file parasite-28-69-s1.pdf]

Supplementary material - Table S1. Nucleotide divergence (p-distance expressed in %) estimated for the 28S rDNA among *Creptotrema* spp. and selected digeneans

		1	2	3	4	5	6	7	8	9	10	11	12	13	14	15	16	17	18	19	20	21	22	23	24	25	26	27	28	29	30	31	32	33	34	35	36	37	38	39	40	41	42	43	44	45	46	47	48	49	50	51	52	53	54	55	56	57	58	59	60	61	62	63	64	65	66	67	68	69	70	71	72	73	74	75	76	77	78	79	80	81	82	83	84	85	86	87	88	89	90	91	92	93	94	95
1	KM871186 *Prosthenhystera caballeroi*
2	EF032690 *Prosthenhystera obesa*	2.6
3	KM871182 *Prosthenhystera oonastica*	2.6	0.0
4	LC215274 *Allocreadium gotoi*	11.5	12.3	12.3
5	GU462125 *Allocreadium isoporum*	12.9	13.7	13.7	5.2
6	GU462126 *Allocreadium isoporum*	12.9	13.7	13.7	5.2	0.0
7	JX977132 *Allocreadium neotenicum*	11.2	12.4	12.4	4.3	4.0	4.0
8	MG262549 *Bunodera mediovitellata*	12.3	13.2	13.2	6.1	7.7	7.7	7.5
9	MG262545 *Bunodera vytautasi*	12.1	12.7	12.7	6.2	8.3	8.3	7.9	2.8
10	GU462123 *Bunodera luciopercae*	12.7	13.5	13.5	6.0	8.1	8.1	7.6	3.4	2.7
11	GU462122 *Bunodera luciopercae*	12.5	13.2	13.2	5.9	8.0	8.0	7.5	3.1	2.6	0.5
12	FR821404 *Crepidostomum farionis*	12.3	13.6	13.6	7.0	9.0	9.0	8.3	7.6	7.2	7.5	7.2
13	FR821403 *Crepidostomum farionis*	12.3	13.6	13.6	7.0	9.0	9.0	8.3	7.6	7.2	7.5	7.2	0.0
14	KY513138 *Crepidostomum farionis*	12.3	13.6	13.6	7.0	9.0	9.0	8.3	7.6	7.2	7.5	7.2	0.0	0.0
15	MN524585 *Acrolichanus auriculatus*	10.7	11.0	11.0	6.0	6.4	6.4	6.0	5.6	5.2	5.0	5.0	5.9	5.9	5.9
16	MN524583 *Acrolichanus auriculatus*	10.7	11.0	11.0	6.0	6.4	6.4	6.0	5.6	5.2	5.0	5.0	5.9	5.9	5.9	0.0
17	FR821398 *Acrolichanus auriculatus*	10.1	10.2	10.2	5.2	6.0	6.0	5.4	5.3	4.8	4.5	4.5	5.2	5.2	5.2	0.6	0.6
18	FR821397 *Acrolichanus auriculatus*	10.1	10.2	10.2	5.2	6.0	6.0	5.4	5.3	4.8	4.5	4.5	5.2	5.2	5.2	0.6	0.6	0.0
19	MN524580 *Acrolichanus auriculatus*	10.4	10.5	10.5	5.4	6.1	6.1	5.6	5.3	4.8	4.6	4.6	5.2	5.2	5.2	0.6	0.6	0.0	0.0
20	MN524579 *Acrolichanus auriculatus*	10.4	10.5	10.5	5.4	6.1	6.1	5.6	5.3	4.8	4.6	4.6	5.2	5.2	5.2	0.6	0.6	0.0	0.0	0.0
21	MT512642 *Creptotrematina batalhensis*	14.6	15.3	15.3	7.3	10.3	10.3	9.2	9.9	8.8	9.4	9.6	9.8	9.8	9.8	9.3	9.3	8.4	8.4	8.7	8.7
22	KF631421 *Creptotrematina aguirrepequenoi*	13.9	14.6	14.6	6.8	10.0	10.0	8.6	8.9	8.2	8.7	8.8	9.3	9.3	9.3	8.7	8.7	7.8	7.8	8.1	8.1	1.6
23	HQ833709 *Creptotrematina aguirrepequenoi*	13.8	14.5	14.5	7.0	10.1	10.1	8.7	9.0	8.3	8.8	9.0	9.4	9.4	9.4	8.9	8.9	8.0	8.0	8.2	8.2	1.7	0.1
24	KJ535505 *Wallinia mexicana*	14.7	16.1	16.1	7.9	11.2	11.2	9.7	9.7	8.9	9.0	8.9	9.2	9.2	9.2	9.0	9.0	8.2	8.2	8.4	8.4	6.1	5.2	5.2
25	KJ535504 *Wallinia mexicana*	14.7	16.1	16.1	7.9	11.2	11.2	9.7	9.7	8.9	9.0	8.9	9.2	9.2	9.2	9.0	9.0	8.2	8.2	8.4	8.4	6.1	5.2	5.2	0.0
26	MW024866 *Wallinia caririensis*	15.1	15.9	15.9	7.9	11.1	11.1	10.3	9.8	8.9	9.5	9.2	9.9	9.9	9.9	8.7	8.7	8.3	8.3	8.3	8.3	6.8	5.5	5.5	4.5	4.5
27	MW024899 *Wallinia caririensis*	14.0	14.7	14.7	7.5	10.3	10.3	9.5	9.3	8.4	8.9	8.5	9.2	9.2	9.2	8.3	8.3	7.6	7.6	7.8	7.8	6.2	5.0	5.0	3.9	3.9	0.1
28	MH520995 *Wallinia brasiliensis*	15.0	15.7	15.7	8.2	11.0	11.0	10.0	9.6	8.7	9.2	8.8	10.2	10.2	10.2	9.2	9.2	8.3	8.3	8.6	8.6	6.4	5.6	5.6	4.0	4.0	2.1	2.0
29	MH997003 *Wallinia anindoi*	15.5	16.4	16.4	9.2	11.8	11.8	10.8	10.2	9.3	9.8	9.4	10.9	10.9	10.9	10.1	10.1	9.2	9.2	9.5	9.5	7.6	6.4	6.4	4.7	4.7	3.1	2.9	1.4
30	MH997004 *Wallinia anindoi*	15.5	16.4	16.4	9.2	11.8	11.8	10.8	10.2	9.3	9.8	9.4	10.9	10.9	10.9	10.1	10.1	9.2	9.2	9.5	9.5	7.6	6.4	6.4	4.7	4.7	3.1	2.9	1.4	0.0
**31**	***Creptotrema conconae* n. sp.**	14.8	15.6	15.6	8.6	11.5	11.5	9.7	10.1	9.9	10.3	10.1	10.4	10.4	10.4	9.8	9.8	9.0	9.0	9.1	9.1	7.1	6.0	6.2	6.8	6.8	5.7	5.6	5.9	6.9	6.9
32	HQ833703 *Wallinia chavarriae*	14.1	15.0	15.0	6.7	10.0	10.0	8.6	8.4	7.6	8.1	7.7	8.9	8.9	8.9	8.2	8.2	7.4	7.4	7.6	7.6	5.2	4.1	4.2	3.4	3.4	3.0	2.6	2.9	3.5	3.5	5.2
33	KF631420 *Creptotrema totonacapanense* **comb. n**	14.9	15.5	15.5	7.5	10.9	10.9	8.6	9.2	8.3	9.0	8.9	9.8	9.8	9.8	8.9	8.9	8.1	8.1	8.2	8.2	6.1	5.1	5.2	5.9	5.9	5.4	5.4	5.6	6.6	6.6	4.7	4.5
34	KF631419 *Creptotrema totonacapanense* **comb. n.**	14.9	15.5	15.5	7.5	10.9	10.9	8.6	9.2	8.3	9.0	8.9	9.8	9.8	9.8	8.9	8.9	8.1	8.1	8.2	8.2	6.1	5.1	5.2	5.9	5.9	5.4	5.4	5.6	6.6	6.6	4.7	4.5	0.0
35	KF631418 *Creptotrema totonacapanense* **comb. n.**	14.9	15.5	15.5	7.5	10.9	10.9	8.6	9.2	8.3	9.0	8.9	9.8	9.8	9.8	8.9	8.9	8.1	8.1	8.2	8.2	6.1	5.1	5.2	5.9	5.9	5.4	5.4	5.6	6.6	6.6	4.7	4.5	0.0	0.0
36	KF631417 *Creptotrema totonacapanense* **comb. n.**	14.9	15.5	15.5	7.5	10.9	10.9	8.6	9.2	8.3	9.0	8.9	9.8	9.8	9.8	8.9	8.9	8.1	8.1	8.2	8.2	6.1	5.1	5.2	5.9	5.9	5.4	5.4	5.6	6.6	6.6	4.7	4.5	0.0	0.0	0.0
**37**	***Creptotrema schubarti* n. sp.**	14.4	15.3	15.3	8.2	11.3	11.3	9.4	9.4	8.6	9.2	9.1	9.6	9.6	9.6	8.9	8.9	8.1	8.1	8.2	8.2	6.3	5.2	5.3	6.1	6.1	5.4	5.1	5.3	6.4	6.4	4.4	4.5	3.4	3.4	3.4	3.4
38	MN822004 *Creptotrema guacurarii* **comb. n**	15.1	15.9	15.9	8.7	11.7	11.7	9.9	9.8	8.9	9.7	9.5	10.0	10.0	10.0	9.2	9.2	8.5	8.5	8.6	8.6	6.6	5.6	5.7	6.4	6.4	5.5	5.5	5.6	6.6	6.6	4.9	4.9	3.8	3.8	3.8	3.8	0.4
**39**	***Creptotrema creptotrema***	15.4	16.1	16.1	8.6	11.6	11.6	9.8	9.6	8.7	9.4	9.2	9.8	9.8	9.8	8.9	8.9	8.1	8.1	8.2	8.2	6.5	5.5	5.6	6.5	6.5	5.5	5.3	5.9	6.7	6.7	5.2	4.5	3.4	3.4	3.4	3.4	2.1	2.5
**40**	***Creptotrema creptotrema***	15.4	16.1	16.1	8.6	11.6	11.6	9.8	9.6	8.7	9.4	9.2	9.8	9.8	9.8	8.9	8.9	8.1	8.1	8.2	8.2	6.5	5.5	5.6	6.5	6.5	5.5	5.3	5.9	6.7	6.7	5.2	4.5	3.4	3.4	3.4	3.4	2.1	2.5	0.0
41	MN822005 *Creptotrema* cf. *stenopteri* **comb. n.**	14.7	15.6	15.6	8.3	10.9	10.9	9.0	8.7	7.9	9.2	9.1	9.7	9.7	9.7	8.2	8.2	8.1	8.1	7.6	7.6	6.4	5.3	5.4	5.8	5.8	4.6	4.9	5.3	6.4	6.4	5.0	4.1	3.1	3.1	3.1	3.1	2.9	2.7	2.8	2.8
42	KF631422 *Creptotrema astyanace* **comb. n.**	13.1	13.9	13.9	6.4	9.5	9.5	7.7	7.7	6.9	7.6	7.5	8.1	8.1	8.1	7.4	7.4	6.7	6.7	6.8	6.8	5.1	4.1	4.2	4.1	4.1	3.7	3.5	4.0	5.0	5.0	3.4	2.9	2.4	2.4	2.4	2.4	2.7	3.0	2.7	2.7	2.0
43	HQ833707 *Creptotrema astyanace* **comb. n.**	13.1	13.9	13.9	6.4	9.5	9.5	7.7	7.7	6.9	7.6	7.5	8.1	8.1	8.1	7.4	7.4	6.7	6.7	6.8	6.8	5.1	4.1	4.2	4.1	4.1	3.7	3.5	4.0	5.0	5.0	3.4	2.9	2.4	2.4	2.4	2.4	2.7	3.0	2.7	2.7	2.0	0.0
44	KX954170 *Creptotrema lobata* **comb. n.**	13.3	13.8	13.8	6.4	9.3	9.3	7.5	7.6	7.0	7.7	7.6	8.4	8.4	8.4	7.3	7.3	6.6	6.6	6.7	6.7	5.2	4.2	4.3	4.4	4.4	3.7	3.7	4.1	5.1	5.1	3.1	3.0	2.3	2.3	2.3	2.3	2.8	3.2	2.8	2.8	1.9	0.3	0.3
45	KX954173 *Creptotrema lobata* **comb. n.**	13.3	13.8	13.8	6.4	9.3	9.3	7.5	7.6	7.0	7.7	7.6	8.4	8.4	8.4	7.3	7.3	6.6	6.6	6.7	6.7	5.2	4.2	4.3	4.4	4.4	3.7	3.7	4.1	5.1	5.1	3.1	3.0	2.3	2.3	2.3	2.3	2.8	3.2	2.8	2.8	1.9	0.3	0.3	0.0
46	KX954171 *Creptotrema lobata* **comb. n.**	13.2	13.8	13.8	6.4	9.3	9.3	7.5	7.6	7.0	7.7	7.6	8.4	8.4	8.4	7.3	7.3	6.6	6.6	6.7	6.7	5.2	4.2	4.3	4.4	4.4	3.7	3.7	4.1	5.1	5.1	3.1	3.0	2.2	2.2	2.2	2.2	2.8	3.2	2.8	2.8	1.9	0.3	0.3	0.0	0.0
47	KX954172 *Creptotrema lobata* **comb. n.**	13.2	13.8	13.8	6.4	9.3	9.3	7.5	7.6	7.0	7.7	7.6	8.4	8.4	8.4	7.3	7.3	6.6	6.6	6.7	6.7	5.2	4.2	4.3	4.4	4.4	3.7	3.7	4.1	5.1	5.1	3.1	3.0	2.2	2.2	2.2	2.2	2.8	3.2	2.8	2.8	1.9	0.3	0.3	0.0	0.0	0.0
48	MH997001 *Creptotrema tica* **comb. n.**	13.4	14.2	14.2	7.3	10.5	10.5	8.5	8.4	7.5	8.5	8.1	8.9	8.9	8.9	7.8	7.8	7.4	7.4	7.5	7.5	5.6	4.6	4.7	5.3	5.3	4.5	4.3	5.0	5.9	5.9	4.1	3.7	2.5	2.5	2.5	2.5	2.0	2.4	2.0	2.0	2.0	1.4	1.4	1.5	1.5	1.5	1.5
49	MH997002 *Creptotrema tica* **comb. n.**	13.4	14.2	14.2	7.4	10.5	10.5	8.5	8.4	7.6	8.5	8.1	8.9	8.9	8.9	7.8	7.8	7.4	7.4	7.5	7.5	5.6	4.6	4.7	5.3	5.3	4.5	4.3	5.0	5.9	5.9	4.1	3.7	2.5	2.5	2.5	2.5	2.0	2.4	1.9	1.9	2.0	1.4	1.4	1.5	1.5	1.5	1.5	0.0
**50**	***Creptotrema megacetabularis* n. sp.**	13.5	14.1	14.1	7.4	10.2	10.2	8.5	8.4	7.4	8.4	8.0	8.7	8.7	8.7	7.7	7.7	7.0	7.0	7.1	7.1	5.8	4.7	4.8	4.9	4.9	4.3	4.1	4.5	5.5	5.5	4.1	3.3	2.8	2.8	2.8	2.8	2.4	2.7	2.4	2.4	1.4	1.5	1.5	1.4	1.4	1.4	1.4	1.3	1.3
**51**	***Creptotrema megacetabularis* n. sp.**	13.4	13.9	13.9	7.4	10.2	10.2	8.5	8.4	7.4	8.4	8.0	8.8	8.8	8.8	7.7	7.7	7.0	7.0	7.1	7.1	5.8	4.8	4.9	5.0	5.0	4.3	4.1	4.5	5.6	5.6	4.0	3.3	2.8	2.8	2.8	2.8	2.4	2.7	2.4	2.4	1.9	1.5	1.5	1.4	1.4	1.4	1.4	1.3	1.3	0.0
**52**	***Creptotrema megacetabularis* n. sp.**	13.4	13.9	13.9	7.4	10.2	10.2	8.5	8.4	7.4	8.4	8.0	8.8	8.8	8.8	7.7	7.7	7.0	7.0	7.1	7.1	5.8	4.8	4.9	5.0	5.0	4.3	4.1	4.5	5.6	5.6	4.0	3.3	2.8	2.8	2.8	2.8	2.4	2.7	2.4	2.4	1.9	1.5	1.5	1.4	1.4	1.4	1.4	1.3	1.3	0.0	0.0
**53**	***Creptotrema megacetabularis* n. sp.**	13.5	14.0	14.0	7.4	10.3	10.3	8.5	8.4	7.4	8.4	8.0	8.7	8.7	8.7	7.7	7.7	7.1	7.1	7.1	7.1	5.8	4.8	4.9	5.0	5.0	4.3	4.1	4.6	5.6	5.6	4.0	3.3	2.8	2.8	2.8	2.8	2.4	2.7	2.4	2.4	1.6	1.5	1.5	1.4	1.4	1.4	1.4	1.3	1.3	0.0	0.0	0.0
54	KT833287 *Paracreptotrema rosenthali*	14.8	15.5	15.5	9.0	11.0	11.0	9.5	9.6	9.0	9.8	9.4	10.7	10.7	10.7	10.1	10.1	9.1	9.1	9.4	9.4	7.0	6.0	6.2	6.1	6.1	6.9	6.1	5.3	6.3	6.3	7.3	5.2	6.0	6.0	6.0	6.0	7.2	7.6	7.2	7.2	6.5	5.1	5.1	5.2	5.2	5.2	5.2	5.8	5.8	5.4	5.5	5.5	5.5
55	KT833288 *Paracreptotrema rosenthali*	15.0	15.7	15.7	9.2	11.3	11.3	9.7	9.8	9.2	10.0	9.6	10.9	10.9	10.9	10.3	10.3	9.3	9.3	9.6	9.6	7.2	6.3	6.4	6.3	6.3	7.1	6.3	5.5	6.6	6.6	7.5	5.4	6.2	6.2	6.2	6.2	7.4	7.8	7.5	7.5	6.7	5.3	5.3	5.4	5.4	5.4	5.4	6.0	6.0	5.7	5.7	5.7	5.7	0.2
56	KT833285 *Paracreptotrema blancoi*	14.4	15.4	15.4	8.0	10.0	10.0	8.5	9.2	7.8	8.2	8.1	9.1	9.1	9.1	8.6	8.6	7.7	7.7	7.9	7.9	6.1	5.5	5.6	4.6	4.6	6.0	5.4	4.8	5.8	5.8	6.8	4.2	5.9	5.9	5.9	5.9	6.3	6.6	6.5	6.5	6.0	4.6	4.6	4.8	4.8	4.8	4.8	5.4	5.4	5.5	5.5	5.5	5.5	4.3	4.5
57	KT833283 *Paracreptotrema blancoi*	14.4	15.4	15.4	8.0	10.0	10.0	8.5	9.2	7.8	8.2	8.1	9.1	9.1	9.1	8.6	8.6	7.7	7.7	7.9	7.9	6.1	5.5	5.6	4.6	4.6	6.0	5.4	4.8	5.8	5.8	6.8	4.2	5.9	5.9	5.9	5.9	6.3	6.6	6.5	6.5	6.0	4.6	4.6	4.8	4.8	4.8	4.8	5.4	5.4	5.5	5.5	5.5	5.5	4.3	4.5	0.0
58	MH159994 *Crepidostomum oschmarini*	11.5	12.3	12.3	5.3	7.2	7.2	6.5	6.2	5.0	6.3	5.9	4.5	4.5	4.5	5.1	5.1	4.5	4.5	4.7	4.7	7.6	7.0	7.1	7.6	7.6	7.2	6.7	7.7	8.3	8.3	8.0	6.4	6.8	6.8	6.8	6.8	7.1	7.5	7.1	7.1	7.1	5.6	5.6	5.7	5.7	5.7	5.7	6.2	6.2	6.0	6.1	6.1	6.1	7.6	7.8	6.4	6.4
59	FR821409 *Crepidostomum metoecus*	11.6	12.3	12.3	5.1	7.1	7.1	6.5	5.8	4.7	5.9	5.6	4.3	4.3	4.3	4.8	4.8	4.2	4.2	4.3	4.3	7.4	6.9	7.0	7.4	7.4	7.0	6.7	7.6	8.2	8.2	7.8	6.3	6.5	6.5	6.5	6.5	7.0	7.4	7.0	7.0	7.0	5.4	5.4	5.3	5.3	5.3	5.3	6.0	6.0	5.7	5.7	5.7	5.7	7.5	7.7	6.3	6.3	0.8
60	KY513145 *Crepidostomum metoecus*	11.6	12.3	12.3	5.1	7.1	7.1	6.5	5.8	4.7	5.9	5.6	4.3	4.3	4.3	4.8	4.8	4.2	4.2	4.3	4.3	7.4	6.9	7.0	7.4	7.4	7.0	6.7	7.6	8.2	8.2	7.8	6.3	6.5	6.5	6.5	6.5	7.0	7.4	7.0	7.0	7.0	5.4	5.4	5.3	5.3	5.3	5.3	6.0	6.0	5.7	5.7	5.7	5.7	7.5	7.7	6.3	6.3	0.8	0.0
61	FR821408 *Crepidostomum metoecus*	11.5	12.2	12.2	5.2	7.2	7.2	6.6	5.7	4.5	5.8	5.4	4.4	4.4	4.4	4.9	4.9	4.3	4.3	4.4	4.4	7.3	6.7	6.9	7.3	7.3	6.9	6.5	7.5	8.1	8.1	7.7	6.1	6.4	6.4	6.4	6.4	6.8	7.2	6.9	6.9	6.9	5.3	5.3	5.2	5.2	5.2	5.2	5.9	5.9	5.5	5.6	5.6	5.6	7.3	7.6	6.1	6.1	0.9	0.1	0.1
62	FR821407 *Crepidostomum metoecus*	11.5	12.2	12.2	5.2	7.2	7.2	6.6	5.7	4.5	5.8	5.4	4.4	4.4	4.4	4.9	4.9	4.3	4.3	4.4	4.4	7.3	6.7	6.9	7.3	7.3	6.9	6.5	7.5	8.1	8.1	7.7	6.1	6.4	6.4	6.4	6.4	6.8	7.2	6.9	6.9	6.9	5.3	5.3	5.2	5.2	5.2	5.2	5.9	5.9	5.5	5.6	5.6	5.6	7.3	7.6	6.1	6.1	0.9	0.1	0.1	0.0
63	FR821406 *Crepidostomum metoecus*	11.5	12.2	12.2	5.2	7.2	7.2	6.6	5.7	4.5	5.8	5.4	4.4	4.4	4.4	4.9	4.9	4.3	4.3	4.4	4.4	7.3	6.7	6.9	7.3	7.3	6.9	6.5	7.5	8.1	8.1	7.7	6.1	6.4	6.4	6.4	6.4	6.8	7.2	6.9	6.9	6.9	5.3	5.3	5.2	5.2	5.2	5.2	5.9	5.9	5.5	5.6	5.6	5.6	7.3	7.6	6.1	6.1	0.9	0.1	0.1	0.0	0.0
64	KF697697 *Paracreptotrematoides heterandriae*	13.5	14.2	14.2	7.2	9.6	9.6	8.4	8.0	7.2	7.4	7.3	7.7	7.7	7.7	7.5	7.5	6.7	6.7	6.9	6.9	5.0	4.4	4.5	4.7	4.7	5.3	4.6	4.8	5.8	5.8	6.5	4.1	5.7	5.7	5.7	5.7	5.8	6.1	5.8	5.8	5.6	4.2	4.2	4.3	4.3	4.3	4.3	5.1	5.1	4.7	4.8	4.8	4.8	5.0	5.2	3.8	3.8	5.9	5.8	5.8	5.7	5.7	5.7
65	KF697696 *Paracreptotrematoides heterandriae*	13.5	14.2	14.2	7.2	9.6	9.6	8.4	8.0	7.2	7.4	7.3	7.7	7.7	7.7	7.5	7.5	6.7	6.7	6.9	6.9	5.0	4.4	4.5	4.7	4.7	5.3	4.6	4.8	5.8	5.8	6.5	4.1	5.7	5.7	5.7	5.7	5.8	6.1	5.8	5.8	5.6	4.2	4.2	4.3	4.3	4.3	4.3	5.1	5.1	4.7	4.8	4.8	4.8	5.0	5.2	3.8	3.8	5.9	5.8	5.8	5.7	5.7	5.7	0.0
66	KT833290 *Pseudoparacreptotrema profundulusi*	14.1	15.0	15.0	7.4	9.4	9.4	8.4	8.6	8.7	8.8	8.7	8.7	8.7	8.7	8.7	8.7	8.1	8.1	8.3	8.3	6.8	6.0	6.1	6.7	6.7	6.7	6.2	6.1	7.2	7.2	7.3	5.5	7.1	7.1	7.1	7.1	7.3	7.6	7.7	7.7	6.7	5.4	5.4	5.3	5.3	5.3	5.3	6.6	6.6	6.0	6.0	6.0	6.1	6.1	6.3	5.3	5.3	6.9	6.7	6.7	6.6	6.6	6.6	4.3	4.3
67	MT180811 *Pseudoparacreptotrema pacificum*	14.6	14.9	14.9	7.3	9.2	9.2	8.8	8.5	8.4	8.6	8.4	8.4	8.4	8.4	8.3	8.3	7.6	7.6	7.8	7.8	7.0	6.0	6.1	6.7	6.7	6.7	6.2	6.5	7.6	7.6	7.3	5.9	6.9	6.9	6.9	6.9	7.6	8.0	7.6	7.6	7.0	5.5	5.5	5.2	5.2	5.2	5.2	6.9	6.9	6.1	6.1	6.1	6.2	6.5	6.7	5.6	5.6	6.6	6.2	6.2	6.1	6.1	6.1	4.4	4.4	1.3
68	MT180810 *Pseudoparacreptotrema pacificum*	14.6	14.9	14.9	7.3	9.2	9.2	8.8	8.5	8.4	8.6	8.4	8.4	8.4	8.4	8.3	8.3	7.6	7.6	7.8	7.8	7.0	6.0	6.1	6.7	6.7	6.7	6.2	6.5	7.6	7.6	7.3	5.9	6.9	6.9	6.9	6.9	7.6	8.0	7.6	7.6	7.0	5.5	5.5	5.2	5.2	5.2	5.2	6.9	6.9	6.1	6.1	6.1	6.2	6.5	6.7	5.6	5.6	6.6	6.2	6.2	6.1	6.1	6.1	4.4	4.4	1.3	0.0
69	MT180818 *Pseudoparacreptotrema pacificum*	14.6	14.9	14.9	7.3	9.1	9.1	8.8	8.5	8.4	8.6	8.4	8.4	8.4	8.4	8.3	8.3	7.6	7.6	7.8	7.8	6.8	6.0	6.1	6.7	6.7	6.7	6.2	6.5	7.6	7.6	7.3	5.9	6.9	6.9	6.9	6.9	7.6	8.0	7.6	7.6	7.0	5.5	5.5	5.2	5.2	5.2	5.2	6.9	6.9	6.1	6.1	6.1	6.2	6.5	6.7	5.7	5.7	6.6	6.2	6.2	6.1	6.1	6.1	4.5	4.5	1.4	0.1	0.1
70	MT180819 *Pseudoparacreptotrema pacificum*	14.6	14.9	14.9	7.3	9.1	9.1	8.8	8.5	8.4	8.6	8.4	8.4	8.4	8.4	8.3	8.3	7.6	7.6	7.8	7.8	6.8	6.0	6.1	6.7	6.7	6.7	6.2	6.5	7.6	7.6	7.3	5.9	6.9	6.9	6.9	6.9	7.6	8.0	7.6	7.6	7.0	5.5	5.5	5.2	5.2	5.2	5.2	6.9	6.9	6.1	6.1	6.1	6.2	6.5	6.7	5.7	5.7	6.6	6.2	6.2	6.1	6.1	6.1	4.5	4.5	1.4	0.1	0.1	0.0
71	MT180822 *Pseudoparacreptotrema pacificum*	14.6	14.9	14.9	7.3	9.1	9.1	8.8	8.5	8.4	8.6	8.4	8.4	8.4	8.4	8.3	8.3	7.6	7.6	7.8	7.8	6.8	6.0	6.1	6.7	6.7	6.7	6.2	6.5	7.6	7.6	7.3	5.9	6.9	6.9	6.9	6.9	7.6	8.0	7.6	7.6	7.0	5.5	5.5	5.2	5.2	5.2	5.2	6.9	6.9	6.1	6.1	6.1	6.2	6.5	6.7	5.7	5.7	6.6	6.2	6.2	6.1	6.1	6.1	4.5	4.5	1.4	0.1	0.1	0.0	0.0
72	MT180823 *Pseudoparacreptotrema pacificum*	14.6	14.9	14.9	7.3	9.1	9.1	8.8	8.5	8.4	8.6	8.4	8.4	8.4	8.4	8.3	8.3	7.6	7.6	7.8	7.8	6.8	6.0	6.1	6.7	6.7	6.7	6.2	6.5	7.6	7.6	7.3	5.9	6.9	6.9	6.9	6.9	7.6	8.0	7.6	7.6	7.0	5.5	5.5	5.2	5.2	5.2	5.2	6.9	6.9	6.1	6.1	6.1	6.2	6.5	6.7	5.7	5.7	6.6	6.2	6.2	6.1	6.1	6.1	4.5	4.5	1.4	0.1	0.1	0.0	0.0	0.0
73	KT833320 *Pseudoparacreptotrema macroacetabulatum*	13.7	14.3	14.3	7.6	10.1	10.1	8.5	8.6	8.0	8.3	8.2	8.4	8.4	8.4	8.1	8.1	7.4	7.4	7.4	7.4	6.7	6.3	6.4	6.9	6.9	6.5	6.0	6.4	7.1	7.1	7.6	5.1	5.9	5.9	5.9	5.9	6.6	7.0	6.4	6.4	5.9	4.9	4.9	5.0	5.0	5.0	5.0	5.2	5.2	5.3	5.3	5.3	5.4	6.5	6.7	5.1	5.1	6.3	6.2	6.2	6.0	6.0	6.0	4.8	4.8	4.0	4.2	4.2	4.1	4.1	4.1	4.1
74	MT180832 *Pseudoparacreptotrema axtlaense*	13.3	14.1	14.1	6.7	9.0	9.0	7.8	7.0	7.1	7.5	7.3	7.9	7.9	7.9	7.6	7.6	6.9	6.9	7.0	7.0	6.1	5.6	5.7	5.6	5.6	5.5	4.8	5.2	6.0	6.0	6.5	4.1	6.0	6.0	6.0	6.0	6.2	6.6	6.3	6.3	5.5	4.3	4.3	4.4	4.4	4.4	4.4	5.3	5.3	4.9	5.0	5.0	5.0	4.8	5.1	4.8	4.8	5.7	5.6	5.6	5.4	5.4	5.4	3.6	3.6	3.2	3.3	3.3	3.2	3.2	3.2	3.2	2.7
75	MT180831 *Pseudoparacreptotrema axtlaense*	13.9	14.7	14.7	6.9	9.2	9.2	8.1	7.2	7.1	7.8	7.7	8.2	8.2	8.2	7.7	7.7	7.2	7.2	7.2	7.2	6.4	5.8	6.0	5.9	5.9	5.5	5.0	5.5	6.2	6.2	6.7	4.3	6.1	6.1	6.1	6.1	6.4	6.8	6.4	6.4	5.1	4.4	4.4	4.5	4.5	4.5	4.5	5.5	5.5	5.1	5.1	5.1	5.1	5.1	5.3	5.0	5.0	6.0	5.8	5.8	5.7	5.7	5.7	3.8	3.8	3.4	3.5	3.5	3.4	3.4	3.4	3.4	2.6	0.0
76	MT180830 *Pseudoparacreptotrema falciformis*	13.7	14.3	14.3	7.3	9.6	9.6	8.4	8.0	7.8	8.3	8.2	8.1	8.1	8.1	8.2	8.2	7.3	7.3	7.5	7.5	6.5	5.9	6.0	5.7	5.7	5.8	5.1	5.3	6.0	6.0	7.0	4.5	6.4	6.4	6.4	6.4	6.6	7.0	6.6	6.6	5.7	4.4	4.4	4.5	4.5	4.5	4.5	5.4	5.5	5.3	5.3	5.3	5.4	5.3	5.5	4.4	4.4	6.1	6.0	6.0	5.9	5.9	5.9	4.1	4.1	3.5	3.7	3.7	3.6	3.6	3.6	3.6	2.4	1.5	1.5
77	MT180829 *Pseudoparacreptotrema falciformis*	13.7	14.3	14.3	7.3	9.6	9.6	8.4	8.0	7.8	8.3	8.2	8.1	8.1	8.1	8.2	8.2	7.3	7.3	7.5	7.5	6.5	5.9	6.0	5.7	5.7	5.8	5.1	5.3	6.0	6.0	7.0	4.5	6.4	6.4	6.4	6.4	6.6	7.0	6.6	6.6	5.7	4.4	4.4	4.5	4.5	4.5	4.5	5.4	5.5	5.3	5.3	5.3	5.4	5.3	5.5	4.4	4.4	6.1	6.0	6.0	5.9	5.9	5.9	4.1	4.1	3.5	3.7	3.7	3.6	3.6	3.6	3.6	2.4	1.5	1.5	0.0
78	MT180825 *Pseudoparacreptotrema falciformis*	13.9	14.4	14.4	7.5	9.7	9.7	8.6	7.9	7.7	8.2	8.1	7.9	7.9	7.9	8.1	8.1	7.2	7.2	7.4	7.4	6.5	5.9	6.0	5.8	5.8	5.9	5.2	5.5	6.1	6.1	7.1	4.7	6.5	6.5	6.5	6.5	6.5	6.9	6.5	6.5	5.8	4.5	4.5	4.6	4.6	4.6	4.6	5.6	5.6	5.4	5.5	5.5	5.5	5.4	5.6	4.5	4.5	6.0	5.9	5.9	5.8	5.8	5.8	4.0	4.0	3.6	3.9	3.9	3.7	3.7	3.7	3.7	2.5	1.6	1.6	0.1	0.1
79	MT180824 *Pseudoparacreptotrema falciformis*	13.9	14.4	14.4	7.5	9.7	9.7	8.6	7.9	7.7	8.2	8.1	7.9	7.9	7.9	8.1	8.1	7.2	7.2	7.4	7.4	6.5	5.9	6.0	5.8	5.8	5.9	5.2	5.5	6.1	6.1	7.1	4.7	6.5	6.5	6.5	6.5	6.5	6.9	6.5	6.5	5.8	4.5	4.5	4.6	4.6	4.6	4.6	5.6	5.6	5.4	5.5	5.5	5.5	5.4	5.6	4.5	4.5	6.0	5.9	5.9	5.8	5.8	5.8	4.0	4.0	3.6	3.9	3.9	3.7	3.7	3.7	3.7	2.5	1.6	1.6	0.1	0.1	0.0
80	MT180826 *Pseudoparacreptotrema falciformis*	14.6	15.1	15.1	8.3	10.2	10.2	8.9	9.0	8.8	9.2	9.0	8.8	8.8	8.8	8.9	8.9	8.1	8.1	8.3	8.3	7.4	6.8	7.0	6.6	6.6	6.2	6.1	6.3	6.7	6.7	8.0	5.4	7.3	7.3	7.3	7.3	7.6	8.0	7.6	7.6	6.6	5.3	5.3	5.4	5.4	5.4	5.4	6.4	6.4	6.2	6.3	6.3	6.3	6.2	6.4	5.1	5.1	6.8	6.7	6.7	6.6	6.6	6.6	5.0	5.0	4.2	4.4	4.4	4.3	4.3	4.3	4.3	3.2	2.3	2.3	0.8	0.8	0.9	0.9
81	MT180828 *Pseudoparacreptotrema falciformis*	13.7	14.3	14.3	7.6	9.6	9.6	8.4	8.3	8.0	8.6	8.4	8.1	8.1	8.1	8.2	8.2	7.3	7.3	7.5	7.5	6.7	6.1	6.3	5.9	5.9	6.1	5.4	5.6	6.0	6.0	7.2	4.8	6.6	6.6	6.6	6.6	6.9	7.3	6.9	6.9	5.9	4.6	4.6	4.8	4.8	4.8	4.8	5.7	5.7	5.5	5.6	5.6	5.6	5.5	5.8	4.7	4.7	6.1	6.0	6.0	5.9	5.9	5.9	4.3	4.3	3.5	3.7	3.7	3.6	3.6	3.6	3.6	2.6	1.7	1.7	0.2	0.2	0.3	0.3	0.6
82	MT180827 *Pseudoparacreptotrema falciformis*	14.3	14.8	14.8	7.7	9.8	9.8	8.5	8.3	8.1	8.5	8.5	8.1	8.1	8.1	8.2	8.2	7.6	7.6	7.6	7.6	7.2	6.6	6.8	6.5	6.5	6.4	6.0	6.1	6.6	6.6	7.3	5.2	6.8	6.8	6.8	6.8	7.3	7.7	7.2	7.2	5.5	4.7	4.7	4.8	4.8	4.8	4.8	5.9	5.9	5.9	5.9	5.9	5.9	5.8	6.1	4.8	4.8	6.2	6.1	6.1	6.1	6.1	6.1	4.5	4.5	3.4	3.8	3.8	3.7	3.7	3.7	3.7	2.5	1.9	1.9	0.2	0.2	0.3	0.3	0.1	0.0
83	JQ425256 *Creptotrema funduli*	13.6	14.8	14.8	7.0	9.7	9.7	8.4	7.4	7.8	7.6	7.5	7.8	7.8	7.8	7.6	7.6	6.8	6.8	7.0	7.0	7.3	6.4	6.3	5.7	5.7	5.8	5.0	5.2	5.8	5.8	6.7	4.6	6.4	6.4	6.4	6.4	6.6	7.0	7.0	7.0	6.3	5.0	5.0	5.1	5.1	5.1	5.1	6.0	6.0	5.5	5.6	5.6	5.6	5.5	5.8	5.6	5.6	6.1	6.0	6.0	5.9	5.9	5.9	4.5	4.5	4.6	5.1	5.1	5.1	5.1	5.1	5.1	5.0	3.7	3.9	4.2	4.2	4.3	4.3	4.8	4.2	4.4
84	EF032694 *Megalogonia ictaluri*	13.0	13.6	13.6	5.4	8.0	8.0	7.2	6.9	6.1	6.2	6.3	7.1	7.1	7.1	6.1	6.1	5.4	5.4	5.6	5.6	4.9	4.3	4.4	4.8	4.8	4.7	4.2	4.6	5.4	5.4	5.4	3.6	5.1	5.1	5.1	5.1	5.3	5.7	5.6	5.6	4.9	3.3	3.3	3.4	3.4	3.4	3.4	4.4	4.4	4.0	4.1	4.1	4.1	5.7	5.9	4.4	4.4	4.4	4.3	4.3	4.2	4.2	4.2	3.3	3.3	3.6	3.6	3.6	3.7	3.7	3.7	3.7	4.0	3.1	3.2	3.6	3.6	3.7	3.7	4.3	3.6	3.9	3.3
85	KF356363 *Crepidostomum affine*	12.0	12.8	12.8	5.9	8.2	8.2	6.6	6.4	5.5	6.0	5.9	6.3	6.3	6.3	5.8	5.8	5.1	5.1	5.2	5.2	5.9	4.9	5.0	5.7	5.7	5.9	5.1	5.6	6.0	6.0	6.6	4.4	5.8	5.8	5.8	5.8	5.6	5.9	5.6	5.6	5.3	4.3	4.3	4.4	4.4	4.4	4.4	4.7	4.7	4.7	4.8	4.8	4.8	5.5	5.7	4.3	4.3	3.7	3.6	3.6	3.5	3.5	3.5	3.7	3.7	4.4	4.6	4.6	4.6	4.6	4.6	4.6	4.1	3.6	3.8	3.6	3.6	3.5	3.5	4.3	3.6	3.9	4.2	3.0
86	KF356372 *Crepidostomum illinoiense*	12.7	13.6	13.6	5.8	8.0	8.0	6.9	5.9	5.6	6.1	6.0	6.8	6.8	6.8	6.1	6.1	5.3	5.3	5.5	5.5	6.0	5.2	5.3	6.0	6.0	5.8	5.2	5.7	6.6	6.6	6.9	4.5	6.2	6.2	6.2	6.2	6.1	6.5	6.2	6.2	5.9	4.6	4.6	4.8	4.8	4.8	4.8	5.5	5.5	5.1	5.1	5.1	5.1	5.1	5.3	5.0	5.0	4.1	4.0	4.0	4.1	4.1	4.1	4.1	4.1	4.0	4.5	4.5	4.5	4.5	4.5	4.5	4.3	3.4	3.6	4.0	4.0	3.8	3.8	4.8	4.2	4.2	4.1	3.3	1.7
87	KF356373 *Crepidostomum auritum*	13.4	13.9	13.9	6.3	8.9	8.9	8.0	6.5	6.1	7.1	6.7	7.4	7.4	7.4	6.7	6.7	6.0	6.0	6.2	6.2	6.5	5.7	5.8	5.5	5.5	5.9	5.2	5.7	6.5	6.5	6.9	4.7	6.3	6.3	6.3	6.3	6.9	7.1	6.9	6.9	5.9	4.9	4.9	4.8	4.8	4.8	4.8	5.7	5.7	4.9	4.9	4.9	4.9	4.9	5.1	4.9	4.9	4.4	4.1	4.1	4.1	4.1	4.1	4.3	4.3	4.5	4.5	4.5	4.5	4.5	4.5	4.5	4.5	3.5	3.7	4.0	4.0	4.1	4.1	4.9	4.2	4.3	3.7	3.3	3.2	2.7
88	KF356374 *Crepidostomum cornutum*	12.7	13.4	13.4	5.6	8.1	8.1	7.1	5.9	5.4	5.9	5.8	6.6	6.6	6.6	6.2	6.2	5.4	5.4	5.6	5.6	5.7	4.9	5.0	4.9	4.9	5.5	4.8	4.8	5.6	5.6	6.4	4.0	5.8	5.8	5.8	5.8	6.2	6.5	6.2	6.2	5.5	4.2	4.2	4.3	4.3	4.3	4.3	5.2	5.2	4.6	4.7	4.7	4.7	3.9	4.1	4.0	4.0	4.1	4.0	4.0	3.9	3.9	3.9	3.5	3.5	3.7	3.7	3.7	3.7	3.7	3.7	3.7	3.9	3.2	3.4	3.3	3.3	3.4	3.4	4.2	3.5	3.7	3.1	2.8	2.1	2.0	1.2
89	KT833278 *Margotrema bravoae*	13.8	14.5	14.5	7.6	9.0	9.0	8.2	7.4	7.2	7.7	7.3	7.2	7.2	7.2	7.8	7.8	7.0	7.0	7.2	7.2	7.5	6.5	6.6	6.7	6.7	7.3	6.4	6.5	7.0	7.0	7.6	5.6	7.5	7.5	7.5	7.5	7.6	8.0	7.6	7.6	6.7	5.6	5.6	5.7	5.7	5.7	5.7	6.2	6.2	5.8	5.8	5.8	5.9	5.3	5.6	5.2	5.2	5.3	5.2	5.2	5.1	5.1	5.1	5.3	5.3	5.2	5.4	5.4	5.4	5.4	5.4	5.4	4.6	4.6	4.9	4.0	4.0	4.1	4.1	4.6	4.0	4.1	4.3	4.1	3.3	3.8	3.1	2.5
90	KT833272 *Margotrema resolanae*	13.2	13.8	13.8	7.0	8.5	8.5	7.7	6.8	6.5	7.3	7.0	7.1	7.1	7.1	6.9	6.9	6.1	6.1	6.3	6.3	7.0	6.0	6.2	6.2	6.2	5.8	5.0	5.7	6.5	6.5	7.1	5.1	7.0	7.0	7.0	7.0	6.9	7.3	7.1	7.1	6.4	5.1	5.1	5.2	5.2	5.2	5.2	5.9	5.9	5.3	5.4	5.4	5.4	5.0	5.2	5.0	5.0	5.0	4.9	4.9	4.7	4.7	4.7	4.4	4.4	4.7	5.0	5.0	5.0	5.0	5.0	5.0	4.4	4.1	4.3	3.7	3.7	3.8	3.8	4.4	3.7	3.9	3.8	3.4	3.0	3.2	2.4	2.0	1.4
91	AF151939 *Dicrocoelium dendriticum*	24.2	25.4	25.4	23.1	23.4	23.4	21.6	22.8	22.2	22.9	22.7	21.8	21.8	21.8	20.0	20.0	19.7	19.7	19.3	19.3	22.9	22.2	22.3	23.7	23.7	23.8	23.0	24.0	24.3	24.3	23.6	22.0	23.3	23.3	23.3	23.3	22.3	22.3	23.1	23.1	22.8	22.2	22.2	22.1	22.1	22.1	22.1	21.9	21.9	21.6	22.3	22.3	21.9	24.3	24.6	22.5	22.5	21.8	21.9	21.9	21.7	21.7	21.7	21.4	21.4	23.7	23.3	23.3	23.3	23.3	23.3	23.3	22.5	23.0	22.9	22.6	22.6	22.4	22.4	23.4	22.6	22.2	24.3	23.0	20.9	22.5	23.3	21.8	23.6	21.9
92	AY222257 *Degeneria halosauri*	21.2	22.2	22.2	19.4	20.8	20.8	20.1	20.4	20.2	20.3	20.2	20.8	20.8	20.8	18.8	18.8	17.8	17.8	18.1	18.1	20.5	20.6	20.8	20.8	20.8	19.7	19.2	20.5	20.8	20.8	21.4	19.6	20.4	20.4	20.4	20.4	20.0	20.5	20.3	20.3	20.1	19.7	19.7	19.9	19.9	19.9	19.9	19.2	19.2	19.2	19.3	19.3	19.3	20.7	21.0	20.8	20.8	18.6	18.8	18.8	18.6	18.6	18.6	20.0	20.0	21.1	20.6	20.6	20.6	20.6	20.6	20.6	19.6	20.3	20.5	20.8	20.8	21.0	21.0	21.8	20.8	21.2	20.2	19.1	18.4	19.7	20.7	19.4	21.8	20.0	18.1
93	KX957729 *Phyllodistomum folium*	23.6	25.1	25.1	26.3	25.1	25.1	24.7	25.9	25.6	25.9	25.7	25.6	25.6	25.6	24.5	24.5	24.1	24.1	24.3	24.3	24.7	23.4	23.6	25.6	25.6	25.9	25.3	26.5	26.8	26.8	25.4	25.2	24.3	24.3	24.3	24.3	23.5	23.9	24.1	24.1	24.4	24.3	24.3	24.4	24.4	24.3	24.3	23.3	23.3	24.5	24.5	24.5	24.5	24.7	25.0	25.0	25.0	24.4	24.8	24.8	24.6	24.6	24.6	25.2	25.2	25.9	25.0	25.0	25.0	25.0	25.0	25.0	25.2	25.3	25.2	25.5	25.5	25.5	25.5	27.0	25.9	26.8	25.9	25.5	23.7	24.8	25.9	24.0	25.0	25.3	24.4	18.2
94	KJ729531 *Phyllodistomum angulatum*	24.7	26.3	26.3	26.2	26.3	26.3	24.8	26.6	26.4	26.8	26.2	26.5	26.5	26.5	25.8	25.8	25.3	25.3	25.5	25.5	25.3	24.1	24.3	26.0	26.0	26.9	25.7	26.8	27.4	27.4	25.9	25.4	25.8	25.8	25.8	25.8	26.2	26.7	26.1	26.1	25.7	25.3	25.3	25.5	25.5	25.4	25.4	25.1	24.9	25.8	25.8	25.8	25.8	24.8	25.1	26.1	26.1	23.8	24.1	24.1	24.3	24.3	24.3	25.9	25.9	26.8	26.0	26.0	26.0	26.0	26.0	26.0	26.3	25.8	25.7	26.5	26.5	26.5	26.5	28.0	26.9	27.7	26.2	26.3	23.7	24.6	25.5	23.8	26.0	25.6	28.8	17.8	14.8
95	AY288828 *Phyllodistomum macrocotyle*	25.2	27.3	27.3	27.9	27.4	27.4	26.3	27.9	27.8	28.4	28.2	28.5	28.5	28.5	27.0	27.0	26.6	26.6	26.9	26.9	26.3	25.1	25.3	27.9	27.9	27.9	27.1	28.4	28.7	28.7	27.5	27.4	27.4	27.4	27.4	27.4	27.8	28.4	27.7	27.7	27.3	26.9	26.9	27.1	27.1	27.1	27.1	26.7	26.5	27.5	27.4	27.4	27.4	26.4	26.7	27.9	27.9	25.4	25.6	25.6	25.8	25.8	25.8	27.5	27.5	29.0	28.1	28.1	28.1	28.1	28.1	28.1	28.6	28.2	28.0	28.5	28.5	28.5	28.5	30.1	29.0	30.2	28.2	28.3	25.5	26.5	27.0	25.7	28.2	27.1	28.1	17.9	14.8	2.8
